# Sumoylation of DNA-bound transcription factor Sko1 prevents its association with nontarget promoters

**DOI:** 10.1371/journal.pgen.1007991

**Published:** 2019-02-14

**Authors:** Veroni S. Sri Theivakadadcham, Benjamin G. Bergey, Emanuel Rosonina

**Affiliations:** Department of Biology, York University, Toronto, Ontario, Canada; The University of Alabama in Huntsville, UNITED STATES

## Abstract

Sequence-specific transcription factors (TFs) represent one of the largest groups of proteins that is targeted for SUMO post-translational modification, in both yeast and humans. SUMO modification can have diverse effects, but recent studies showed that sumoylation reduces the interaction of multiple TFs with DNA in living cells. Whether this relates to a general role for sumoylation in TF binding site selection, however, has not been fully explored because few genome-wide studies aimed at studying such a role have been reported. To address this, we used genome-wide analysis to examine how sumoylation regulates Sko1, a yeast bZIP TF with hundreds of known binding sites. We find that Sko1 is sumoylated at Lys 567 and, although many of its targets are osmoresponse genes, the level of Sko1 sumoylation is not stress-regulated and the modification does not depend or impinge on its phosphorylation by the osmostress kinase Hog1. We show that Sko1 mutants that cannot bind DNA are not sumoylated, but attaching a heterologous DNA binding domain restores the modification, implicating DNA binding as a major determinant for Sko1 sumoylation. Genome-wide chromatin immunoprecipitation (ChIP-seq) analysis shows that a sumoylation-deficient Sko1 mutant displays increased occupancy levels at its numerous binding sites, which inhibits the recruitment of the Hog1 kinase to some induced osmostress genes. This strongly supports a general role for sumoylation in reducing the association of TFs with chromatin. Extending this result, remarkably, sumoylation-deficient Sko1 binds numerous additional promoters that are not normally regulated by Sko1 but contain sequences that resemble the Sko1 binding motif. Our study points to an important role for sumoylation in modulating the interaction of a DNA-bound TF with chromatin to increase the specificity of TF-DNA interactions.

## Author summary

Transcription factors bind the genome at specific sites to control the expression of target genes. Although the DNA sequence of these sites is critical for determining where they bind, additional factors must act to ensure that only appropriate sites remain bound and regulated by transcription factors. Here we demonstrate that SUMO post-translational modification functions in transcription factor binding site selection. We show that a yeast transcription factor, Sko1, becomes sumoylated after it binds DNA, and that the modification reduces the interaction of Sko1 with DNA. We propose that this is important to ensure that Sko1 remains associated only with actual Sko1 genomic binding sites. Indeed, we find that a mutant form of Sko1 that cannot be sumoylated binds hundreds of sites on the genome that are not normally bound by Sko1. Most of these additional binding sites contain sequences that somewhat resemble the normal Sko1 binding site sequence, suggesting that sumoylation increases the stringency of Sko1 binding site selection. As numerous transcription factors are known to be targets of SUMO modification, our work suggests a general role for sumoylation in promoting specificity of binding to genomic sites in eukaryotic cells.

## Introduction

Sumoylation is an essential eukaryotic post-translational modification that functions in many, predominantly nuclear, cellular processes, such as DNA repair and transcription, by regulating target protein localization, stability, or interactions with other proteins or with chromatin [1–5]. The modification involves the covalent attachment of a ~12-kDa SUMO (*S*mall *U*biquitin-like *Mo*difier) peptide to specific lysine residues on substrate proteins through a three-enzyme cascade that is analogous to ubiquitination [1]. In contrast to ubiquitination, however, the sole SUMO E2 conjugating enzyme Ubc9 can recognize its substrates via the consensus sequence ΨKxD/E, where Ψ is a hydrophobic residue, K is the modified lysine and x is any amino acid [6]. Although thousands of proteins have been identified as potential SUMO targets, modification levels of individual proteins are typically low and can be controlled by the desumoylating action of SUMO-specific isopeptidases (SUMO proteases), including the SENP family in mammals, and Ulp1 and Ulp2 in budding yeast [3,7,8]. On a global level, cellular sumoylation can be coordinately regulated, which is exemplified by the SUMO stress response, a rapid increase in overall SUMO conjugation that is observed in both yeast and mammalian cells in response to various stress conditions, including temperature, oxidative, and osmotic stress [4,9–12].

Chromatin immunoprecipitation (ChIP) analyses, both genome-wide and on individual genes, have demonstrated that sumoylated proteins are detected specifically at promoter regions of constitutively active and induced genes, suggesting that the modification is important for regulating early steps of transcription [13–17]. Supporting this, proteomics studies have identified subunits of the general transcription factors (GTFs), RNA Polymerase II (RNAP II), and Mediator as SUMO conjugates in yeast, Drosophila, and human cells [18]. Moreover, one of the largest groups of SUMO substrates, with over 300 substrates identified in human SUMOylome analyses, is sequence/gene-specific transcription factors (TFs) [3,19]. Numerous studies have characterized the role of sumoylation on individual TFs, and in most cases the modification is associated with a repressive effect on transcription of target genes [5,19–21]. For example, upon sumoylation, NFAT, Elk-1 and MafG interact with histone deacetylases (HDACs) to condense DNA and restrict access of the transcription machinery, whereas sumoylation of the Forkhead Box TF FoxM1b promotes its cytoplasmic retention and ubiquitin-mediated degradation, thereby limiting its access to target genes [22–25]. Why a transcriptionally repressive mark like SUMO is enriched at promoter regions of transcriptionally active genes remains unknown.

Providing a possible explanation, studies on two basic leucine zipper (bZIP) motif-containing TFs, yeast Gcn4 and human FOS (c-Fos), showed that promoter-associated sumoylation is important for regulating their occupancy levels [26–28]. DNA-bound Gcn4 and FOS are sumoylated during active transcription, and the modification promotes their clearance from DNA. This limits the association of these TFs with their target genes once they are activated, which likely serves to prevent excessive gene expression. Consistent with this, ChIP studies have shown that sumoylation modulates the chromatin occupancy of multiple other TFs, including human TFs FOXA1, MITF, c-Maf, and the androgen and glucocorticoid nuclear receptors [29–33]. How sumoylation affects TF occupancy is not always known, but in some cases SUMO modifications directly affect DNA binding. For example, modification of SP1, specifically by the SUMO2 isoform, reduces its DNA binding activity in an in vitro assay [34]. These studies have led to speculation that sumoylation has a general role in controlling the interaction of TFs with their target sites on chromatin [19]. This can serve to regulate the expression levels of target genes, but another possible function for SUMO-mediated modulation of TF-chromatin interactions is in binding site selection. Multiple factors, in addition to DNA sequence, play a role in determining what genomic sites are recognized and bound by TFs, including genomic context, DNA modifications, the nature of flanking DNA sequences, and interaction with cofactors [35,36]. However, it is not known whether sumoylation plays a role in regulating binding site selection for TFs across eukaryotes.

To address this, we examined how sumoylation regulates the bZIP TF Sko1 (*S*uppressor of *K*inase *O*verexpression-*1*) from the budding yeast, *Saccharomyces cerevisiae*. Sko1 was identified as a putative SUMO target in multiple proteomics screens, suggesting that its activity or association with chromatin is regulated by sumoylation [37–39]. It was first characterized as a repressor of cAMP response element (CRE)-containing genes, such as *HIS3* and *ENA1*, that are induced after exposure to osmotic stress [40–43]. Repression by Sko1 involves recruitment of the Tup1/Cyc8 corepressor complex, and relief of repression occurs during osmotic stress as a result of phosphorylation by the activated MAP kinase Hog1 [40,41,44–47]. In addition to functioning as a repressor, Sko1 was subsequently shown to be involved in the induction of some stress response genes, through the Hog1-dependent recruitment of the SAGA and SWI/SNF nucleosome remodelers to promoter regions bound by Sko1/Tup1/Cyc8 complexes [45,47,48]. Genome-wide analyses showed that Sko1 is constitutively associated with >250 gene promoters and that osmotic stress results in a general redistribution of Sko1, with some genomic sites showing stress-dependent gain or loss of Sko1, while occupancy at other sites remains unchanged [49–51]. Nonetheless, many Sko1 binding sites are not associated with genes specifically involved in the osmotic stress response, and *sko1Δ* cells show no growth defect in high osmolarity medium, suggesting that the TF plays a wider role in gene regulation [45,49].

Here, we demonstrate that Sko1 is indeed a SUMO target and that the modification functions primarily in preventing Sko1 from associating with nonspecific sites on the genome. We identify Lys 567 as the major SUMO modification site on the TF and show that Sko1 sumoylation is a constitutive modification whose levels do not significantly change under different growth conditions, including osmotic stress. DNA binding activity is necessary for Sko1 sumoylation in vivo, implying that the modification takes place after Sko1 associates with chromatin. Significantly, our genome-wide analysis shows that a sumoylation-deficient form of Sko1 shows increased occupancy levels at its binding sites and is found at numerous additional promoter regions across the genome, when compared with sumoylatable Sko1. Intriguingly, although the consensus Sko1 binding motif is largely absent from these additional sites, most contain sequences that resemble the motif. Taken together, our results imply that a key function for sumoylation is in controlling the association of a DNA-bound TF with chromatin to increase its binding site specificity.

## Results

### Sko1 is sumoylated at Lys 567 in yeast grown in normal conditions

**Fig 1:**
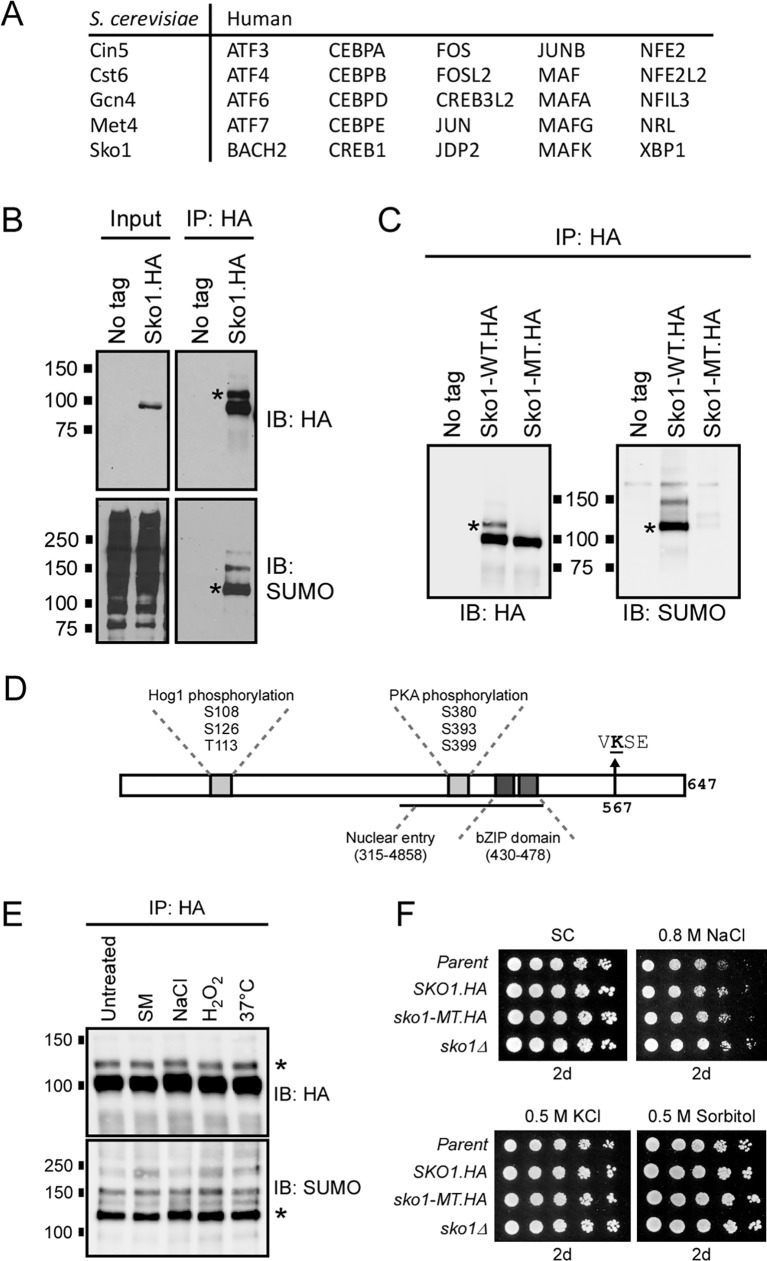
Sko1 is constitutively sumoylated at Lys 567. **(A)** List of yeast and human bZIP motif-containing proteins that have been identified as putative SUMO targets in published proteomics studies. **(B)** Lysates from yeast strains expressing 6xHA-tagged Sko1 from its chromosomal locus, or a strain with no HA-tagged proteins (*No tag*), were examined by HA IP and immunoblot analysis with HA and SUMO antibodies. Inputs represent approximately 5–10% of the IPed material. **(C)** A yeast strain was generated with a chromosomal *sko1* K567R mutation (Sko1-MT.HA) and used for IP-immunoblot analysis, as in *(B)*, alongside *No tag* and Sko1-WT.HA controls. **(D)** Schematic of the Sko1 protein indicating bZIP DNA binding domain, regions targeted by Hog1 and PKA kinases, region required for nuclear entry [54], and the primary site of sumoylation, Lys 567, which is found within a SUMO consensus motif, VKSE. **(E)** IP-immunoblot analysis of lysates from the *SKO1-WT*.*HA* strain grown in SC medium (“Untreated”), or SC medium treated for 20 min with 0.5 μg/mL sulfometuron methyl (SM, amino acid starvation), 0.4 M NaCl (osmotic stress), 1 mM H_2_O_2_ (oxidative stress), or incubated at 37°C (temperature stress), as in *(B)*. **(F)** Spot assay comparing growth of indicated strains in various types of osmotic stress. Approximately 10,000 cells were spotted on the left-most position and serial five-fold dilutions were spotted on adjacent spots to the right in each panel. Media plates were imaged after two days. Asterisks (*) indicate position of the major (mono-) sumoylated form of Sko1 in each immunoblot.

Previous studies demonstrated that for two different bZIP TFs, Gcn4 in budding yeast and human FOS, sumoylation acts to restrict their association with DNA thereby preventing excessive expression of target genes [26–28]. We examined published lists of sumoylated proteins that were generated through proteomics analyses and identified 28 additional bZIP motif-containing TFs that are probable SUMO targets, four in *S*. *cerevisiae* and 24 human proteins (Fig 1A) [4,8]. This represents almost half of known bZIP TFs in these species and suggests that sumoylation is a common mechanism of regulating their functions. To explore this, we selected for further study the yeast bZIP TF Sko1 which was identified as a SUMO target in large-scale studies, but the effects of its sumoylation have not yet been reported [37–39].

A yeast strain was generated that expresses a C-terminal 6xHA-tagged form of Sko1 (Sko1.HA) from its natural locus and we confirmed that the presence of the tag does not affect cell growth under normal and osmotic stress conditions (S1A Fig). Cell lysates were analyzed using the same procedures that were previously used for examining sumoylated proteins in yeast, i.e. HA immunoprecipitation (IP) followed by HA and SUMO immunoblotting (see *Materials and Methods*) [27,28,52]. Protein bands of the molecular weights expected for sumoylated Sko1 were detected in the SUMO immunoblot, including a “ladder” of bands that is typical for proteins that are multi- or poly-sumoylated, confirming that Sko1 is sumoylated (Fig 1B). Supporting that the sumoylated species detected in the immunoblots correspond to modified Sko1 specifically, we repeated the analysis using protein samples that were prepared under denaturing conditions (i.e. precipitation with trichloroacetic acid, TCA), which again showed bands of the expected size in the SUMO immunoblot (S1B Fig). These results confirm the observation made in multiple proteomics studies that Sko1 is sumoylated in yeast grown in normal conditions [37–39].

We analyzed the polypeptide sequence of Sko1 using a SUMO-site prediction tool, GPS-SUMO [53], and identified Lys 567, which is within a SUMO consensus motif, as the most likely site of SUMO modification. To test this, we generated a yeast strain expressing only a K567R-mutant form of Sko1.HA from the *SKO1* locus (hereafter referred to as Sko1-MT), and examined its sumoylation by IP-immunoblot. As shown in Fig 1C, Sko1-MT is expressed at normal levels, but the mutation virtually abolishes its sumoylation, indicating that Lys 567 is the major site of sumoylation on the protein. This residue lies in the C-terminal part of the protein, significantly downstream of the bZIP DNA binding motif, the Hog1 and PKA phosphorylation sites, and the region of the protein required for nuclear entry, and therefore defines a novel regulatory region on Sko1 (Fig 1D) [54]. A higher molecular weight form of Sko1 that is detectable in HA immunoblots of Sko1-WT (asterisks in Figs 1B, 1C, 1E and S1B), but is not present in the Sko1-MT IP lane in Fig 1C, corresponds in size to monosumoylated Sko1, implying that both unmodified Sko1 and its major sumoylated form can be detected on the same HA immunoblot. Taking advantage of this, we used densitometry to measure the intensity of bands corresponding to unmodified and monosumoylated Sko1 and determined that at least 13% of Sko1 is sumoylated in normally growing yeast. Nonetheless, sumoylation of Sko1 is not required for cell viability as the *sko1-MT* strain grew as well as strains expressing Sko1-WT on rich (YPD) or synthetic complete (SC) media (S1C Fig). This result is not surprising since *SKO1* itself is not required for normal yeast growth (e.g. *sko1Δ* in S1C Fig and [44]), but our data indicates that, at any given time, a significant fraction of Sko1 polypeptides are modified by sumoylation.

### Sko1 sumoylation is not affected by osmotic stress

To examine whether sumoylation might regulate Sko1 in response to high osmolarity or other types of stress, the *SKO1-WT* strain was grown in different conditions, and the level of Sko1 sumoylation was determined in each condition by IP-immunoblot analysis. As shown in Figs 1E and S1D, compared to growth in normal medium (“untreated”), Sko1 sumoylation levels were essentially unchanged after exposure to osmotic, oxidative, or temperature stress, or during amino acid starvation (through addition of sulfometuron methyl, SM, to growth medium) [55]. This indicates that sumoylation of Sko1 does not occur as part of a stress response but that the modification regulates Sko1 constitutively. Cellular levels of Sko1 in *SKO1-WT* and *sko1-MT* strains were approximately the same under normal growth conditions, and remained constant during osmotic stress, implying that sumoylation does not act to regulate Sko1 stability or abundance (S1E Fig). Moreover, the *sko1-MT* strain grew as well as the strain expressing Sko1-WT on a variety of osmotic stress media, indicating that Sko1 sumoylation is not required for cell survival during osmotic stress (Fig 1F). However, Sko1 itself is not required for survival during osmotic stress (*sko1Δ* in Fig 1F and [45]), reflecting that its role in regulating the transcription of target stress response genes has more subtle consequences, and that the effects of sumoylation on Sko1 function are likely intricate (see below) [48].

### Sko1 sumoylation does not depend on or influence its phosphorylation

**Fig 2:**
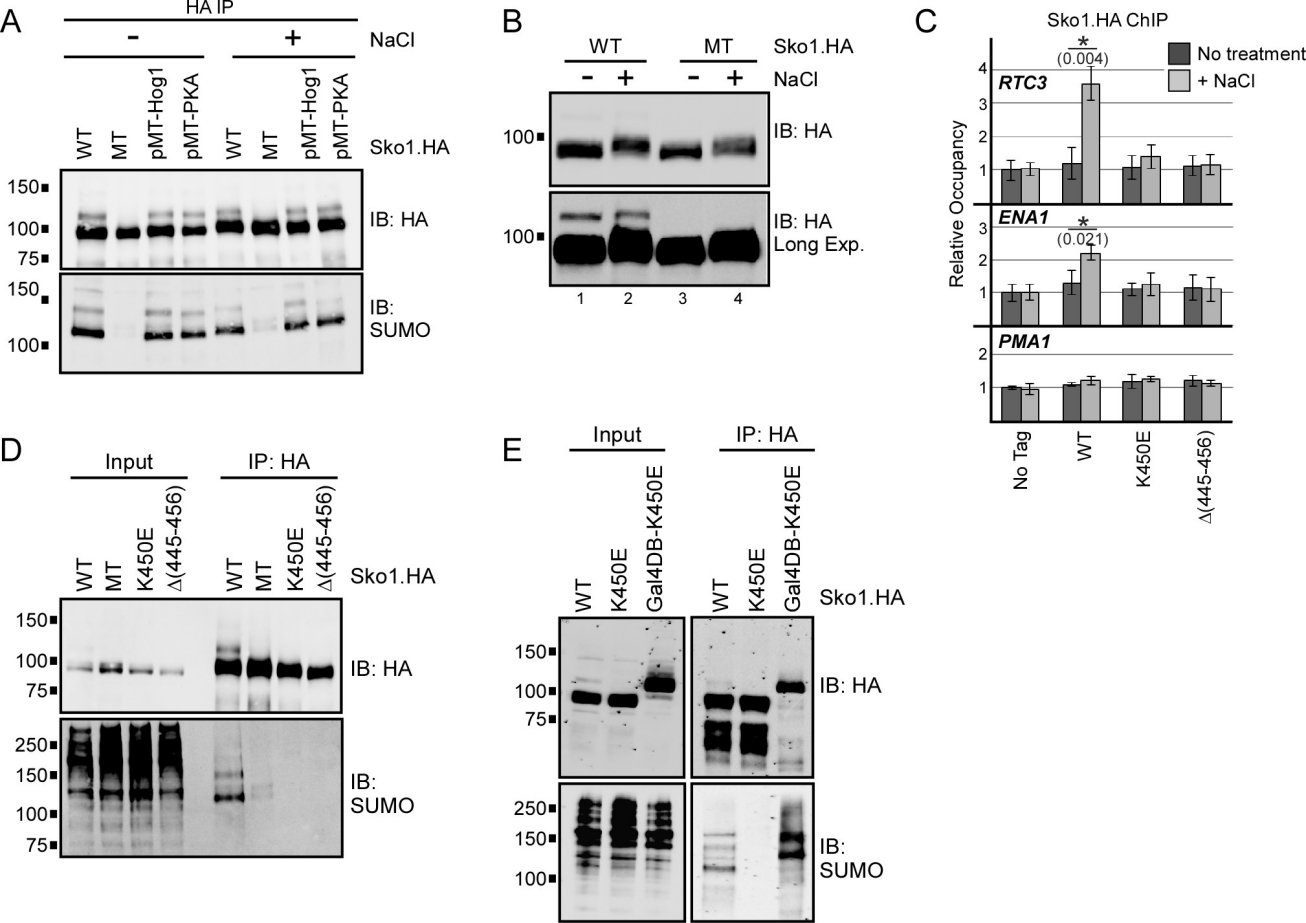
Sko1 sumoylation shows no interdependence with phosphorylation but requires DNA binding. **(A)** Yeast strains expressing WT, MT, Hog1 kinase site-mutant (“Sko1(pMT.Hog1)”; S108,126A, T113A), or PKA kinase site-mutant (“Sko1(pMT.PKA)”; S380,393,399A) forms of Sko1.HA were grown in untreated medium or with 0.4 M NaCl for 10 min. Lysates were then prepared and subjected to IP-immunoblot analysis as in Fig 1B. **(B)** HA immunoblot analysis of lysates from *SKO1-WT*.*HA* and *sko1-MT*.*HA* strains that were grown in untreated medium, or with 0.4 M NaCl for 10 min. A longer exposure is shown to highlight the differential migration of the sumoylated (higher molecular weight) form of Sko1.HA in the first two lanes. **(C)** Yeast strains expressing no HA-tagged proteins or WT, K450E or Δ(445–456) forms of Sko1.HA, were grown in normal conditions or treated with 0.4 M NaCl for 5 min, then cross-linked for HA ChIP analysis. qPCR was performed on promoter regions of two Sko1 target genes, *RTC3* and *ENA1*, and a non-Sko1 target gene, *PMA1*. Error bars represent standard deviation of three independent experiments. Asterisks (*) indicate that the compared data pairs are statistically different (*P* < 0.05; see *Materials and Methods*). **(D)** IP-immunoblot analysis of lysates from yeast strains expressing indicated forms of Sko1.HA, performed as in Fig 1B. **(E)** IP-immunoblot analysis of lysates from strains expressing WT, K450E, or Gal4-DNA binding domain-fused Sko1-K450E (GalDB-K450E) forms of Sko1.HA.

Many SUMO modifications show codependence or interference with other types of post-translational modifications, including phosphorylation, on the same protein [19,56]. To determine whether prior phosphorylation by Hog1 or PKA is required for Sko1 sumoylation, we generated yeast strains expressing mutant forms of Sko1.HA, including Sko1(pMT.Hog1), which contains Ala substitutions at the three Hog1 target residues (Ser 108, Ser 126, and Thr 113), and Sko1(pMT.PKA), which has Ala substitutions at the three PKA target Ser residues (380, 393, and 399) [44]. The strains were then grown untreated or treated with NaCl for 10 min prior to the preparation of lysates and subsequent examination by IP-immunoblot. As shown in Fig 2A, normal levels and patterns of Sko1 sumoylation were observed in the SUMO immunoblot analysis of both the Sko1(pMT.Hog1) and Sko1(pMT.PKA) forms of Sko1.HA. This indicates that sumoylation of Sko1 occurs independently of its prior phosphorylation by Hog1 or PKA.

Next we examined whether sumoylation can influence subsequent phosphorylation of Sko1 by Hog1, which occurs in response to osmotic stress [44]. HA immunoblot analysis of Sko1-WT from cells grown in osmotic stress showed a mobility shift that is consistent with its phosphorylation and similar to a mobility shift that was previously reported (Fig 2B) [44]. The NaCl-dependent shift occurred for both the prominent band in the immunoblot, which corresponds to unsumoylated Sko1, as well as for the monosumoylated form of Sko1-WT (compare lanes 1 and 2 in upper and lower panels of Fig 2B). Sko1-MT also showed a NaCl-dependent shift in migration during osmotic stress (compare lanes 3 and 4). To examine this further, we repeated the HA immunoblot analysis using a phosphate-binding compound present in the acrylamide mix that enhances detection of phosphorylated protein isoforms during SDS-PAGE (“Phos-tag”) [57]. As shown in S1F Fig, prominent higher-molecular weight bands appeared after treatment of either *SKO1-WT* or *sko1-MT* strains with NaCl (“Sko1-P”), which we attribute to Hog1-mediated phosphorylation since this shift was not observed in a *hog1Δ* strain. Together, these analyses indicate that sumoylation is not a pre-requisite for Sko1 phosphorylation during osmotic stress but suggests that both unsumoylated and sumoylated Sko1 can be phosphorylated by Hog1.

### DNA binding is necessary and sufficient for Sko1 sumoylation

To investigate whether Sko1 becomes sumoylated prior to, or after binding target DNA sequences, we tested if its DNA binding activity is necessary for the modification to take place. Lys 450 of Sko1 is at a conserved position in the bZIP motif, and a Lys-to-Glu mutation of the corresponding Lys in the human bZIP TF CREB1 abolished its ability to bind DNA [43,58]. We constructed yeast strains that expresses an analogous K450E mutant form of Sko1.HA, or Sko1.HA with a 12-amino acid residue deletion surrounding Lys 450, Δ(445–456). To confirm that these mutant forms of Sko1 are indeed defective in DNA binding, we performed ChIP analysis at the promoter regions of the *RTC3* and *ENA1* genes, both of which are bound by Sko1 during osmotic stress [49,50], and *PMA1*, which is not targeted by Sko1 [48]. As expected, treatment of the *SKO1-WT* strain with NaCl led to rapid recruitment of Sko1 to the *RTC3* and *ENA1* promoters, but not to the promoter of *PMA1* (Fig 2C). For both Sko1-K450E and Sko1-Δ(445–456), however, no recruitment was detected, indicating that both forms of Sko1 are indeed defective in DNA binding. WT, MT, and the DNA-binding-deficient forms of Sko1.HA were then examined by IP-immunoblot, and, as shown in Fig 2D, even though the K450E and Δ(445–456) forms were expressed and IPed at levels comparable to Sko1-WT, neither showed a signal on the SUMO immunoblot. This indicates that Sko1 that is not able to bind DNA is not sumoylated and suggests that the modification takes place only after the TF binds to its target DNA sites.

To determine whether DNA binding itself can trigger Sko1 sumoylation, we constructed an expression plasmid that generates the DNA binding-deficient form of Sko1.HA (K450E) fused to the DNA binding domain of the transcription activator Gal4 (construct Gal4DB-K450E). Strikingly, when introduced into a yeast strain that contains multiple Gal4 binding sites, the fusion protein showed high levels of sumoylation (Fig 2E). This indicates that DNA binding itself can restore SUMO modification to the Sko1-K450E mutant, and strongly implies that DNA binding is a major determinant for Sko1 sumoylation.

### Genome-wide identification of Sko1-WT and Sko1-MT binding sites

**Fig 3:**
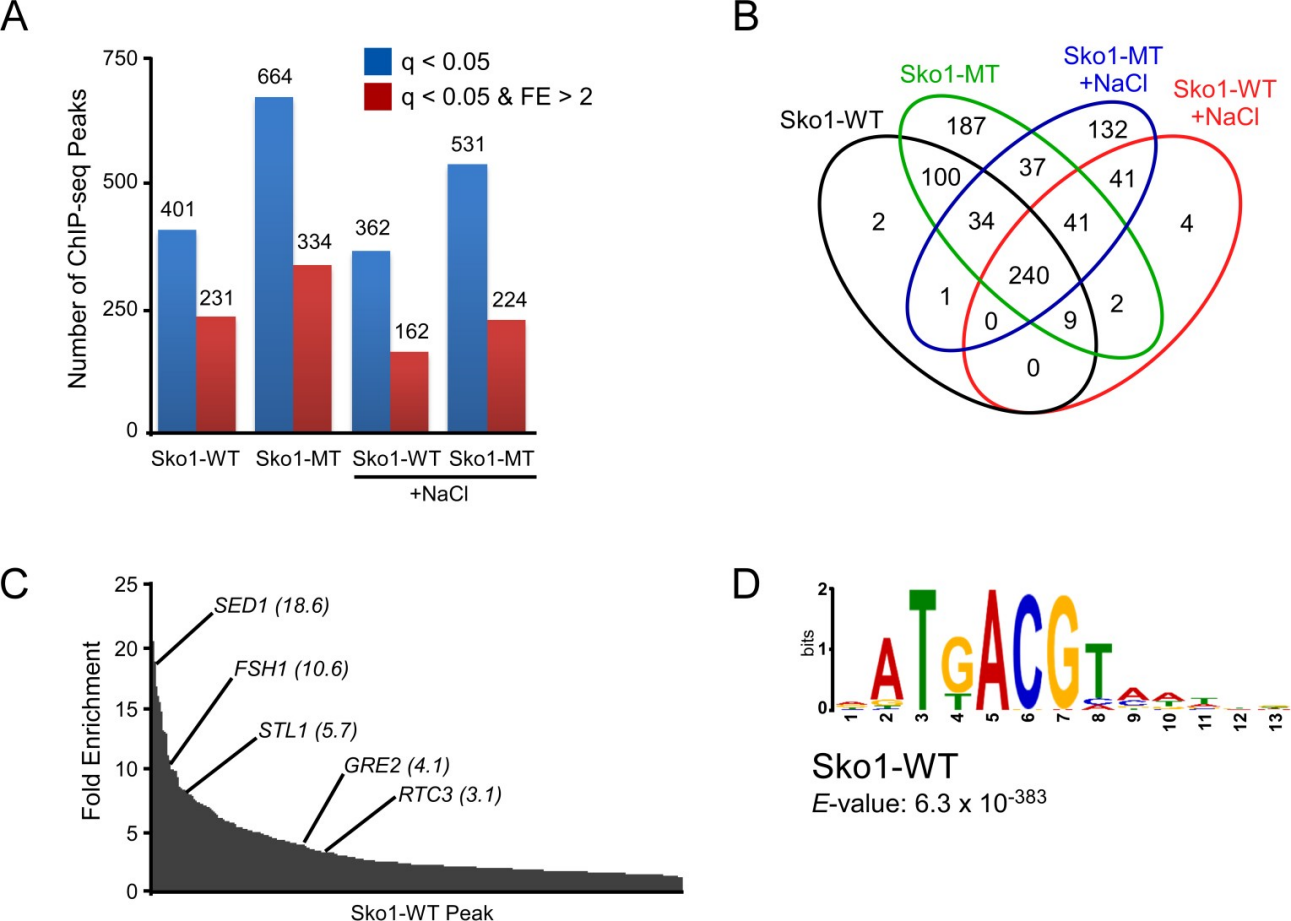
Binding site analysis from Sko1-WT and Sko1-MT ChIP-seq experiment. **(A)** Number of binding sites (peaks) identified from Replicate 1 of ChIP-seq analysis of *SKO1-WT*.*HA* and *sko1-MT*.*HA* strains, either untreated or treated with 0.4 M NaCl for 5 min, with a *q-*value less than 0.05 (blue bars). Subset of identified peaks having a *q*-value less than 0.05 and a fold enrichment (FE) value greater than 2 are also indicated, in red. **(B)** Venn diagram showing number of peaks (*q* < 0.05) shared among the four samples tested. When generating Venn diagrams throughout this study, if two peaks in one sample both overlap with the same peak in another sample they are counted as a single overlapping peak. This results in slightly fewer total peaks for each sample in the Venn diagram compared with the number of identified peaks listed in S3 and S4 Tables and in panel (*A)*. **(C)** Distribution of fold enrichment values for the peaks identified in the untreated Sko1-WT sample, with peaks of select known Sko1 target genes indicated with FE values shown in brackets. **(D)** Motif discovery analysis, using MEME tool, of the sequences surrounding the summit of the peaks from the untreated Sko1-WT sample, identified the indicated consensus sequence.

To explore whether sumoylation regulates the association of Sko1 with chromatin at its binding sites genome-wide, we performed HA ChIP followed by next-generation sequencing (ChIP-seq) for both *SKO1-WT* and *sko1-MT* strains, under normal growth and after exposure to osmotic stress (0.4 M NaCl for 5 min). Sko1 binding sites (peaks) were identified, using the MACS2 software tool, through detection of genomic regions with statistically significant Sko1 occupancy in IPed samples relative to background levels from input samples (statistical significance cut-off of *q* < 0.05). Numerous peaks were identified for both Sko1-WT and Sko1-MT in both untreated and osmotic stress-treated (+NaCl) samples (Fig 3A and 3B and S3 Table), including peaks near many known Sko1-regulated genes (Fig 3C and see S5 Table). NaCl-treated samples had somewhat fewer identified peaks than untreated samples for both Sko1-WT and Sko1-MT, which likely reflects the osmotic stress-associated binding dynamics of Sko1 in which its levels are reduced on some targets [49–51]. Consistent with previous genome-scale binding studies, occupancy levels of Sko1-WT range widely among its binding sites, with about half showing relatively high occupancy levels (at least a two-fold enrichment in read density compared to input, FE > 2; Fig 3A and 3C), including binding sites for some known Sko1 target genes, as indicated in Fig 3C [48–51]. Supporting the effectiveness of this ChIP-seq experiment, we compared it with a previous Sko1 ChIP-seq study using the ChIPPeakAnno analysis toolkit and found that 80% of the peaks in our Sko1-WT set were also identified in that study [50]. Furthermore, a search for recurring sequences in the Sko1-WT peak set from our analysis produced the previously reported Sko1 binding motif, ATGACGT, with very high confidence (Fig 3D) [50,59].

### Sko1-MT binds more promoter regions than Sko1-WT

Intriguingly, the ChIP-seq analysis showed that Sko1-MT binds dramatically more sites than Sko1-WT, in both untreated and +NaCl samples (66% and 47% more peaks, respectively; Fig 3A and 3B, and S3 Table). To confirm this observation, we performed an independent ChIP-seq replicate experiment (Replicate 2), which again showed more binding sites for Sko1-MT than Sko1-WT in untreated and +NaCl conditions (S2A and S2B Fig). Overall, Replicate 2 showed consistently lower normalized read counts (i.e. occupancy levels) than Replicate 1 and it consisted of many peaks of low fold enrichment that were largely absent from Replicate 1 (S2A and S2C Fig). As we did not produce an input control set for Replicate 2, this might reflect that peak assignment normalization was performed by other methods for this replicate (see *Materials and Methods*). Nonetheless, a large number of overlapping peaks were identified in each analysis between the two replicates (S2C Fig, left and S3 Table), and, when only peaks from Replicate 2 that have high fold enrichment (FE > 2) were considered, 75% to 91% were found to overlap with peaks from Replicate 1 (S2C Fig, right). Importantly, each replicate identified more peaks bound by Sko1-MT compared with Sko1-WT, regardless of whether yeast were grown in normal or osmotic conditions (compare Fig 3A with S2A Fig). To increase confidence in our results, the peak analyses described below were performed using only binding sites that were identified in both replicates for each sample (“overlapping peak sets”). Notably, the overlapping peak sets also showed dramatically higher numbers of peaks for Sko1-MT than for Sko1-WT in untreated and +NaCl conditions (S2D and S2E Fig). Lists of peaks identified in each replicate and in the overlapping peak sets are presented in S3 and S4 Tables, respectively.

**Fig 4:**
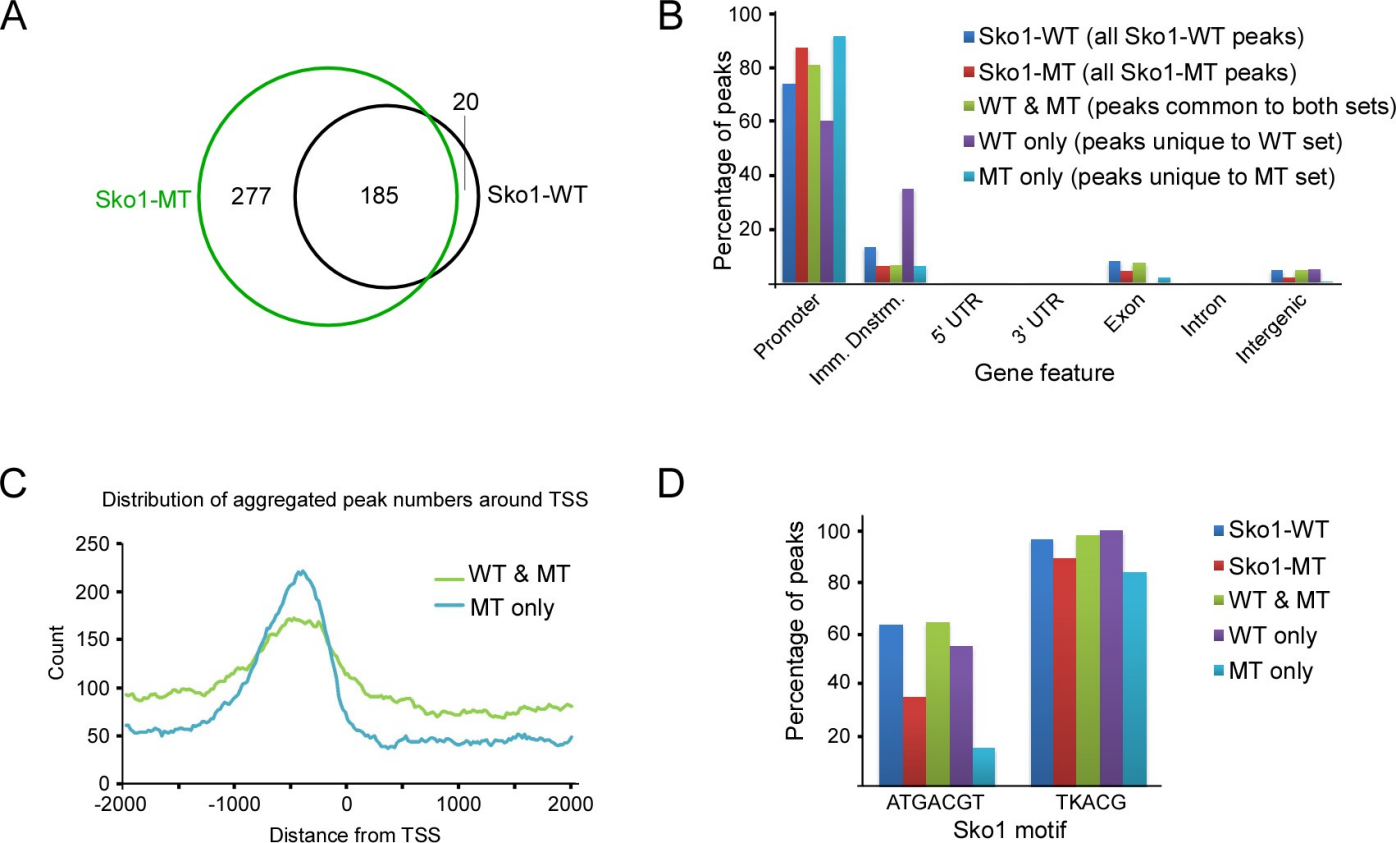
Sko1-MT binds dramatically more promoter regions than Sko1-WT. **(A)** Venn diagram showing number of common and unique peaks from the untreated Sko1-WT and Sko1-MT ChIP-seq analyses. Peaks identified in both ChIP-seq replicates (i.e. overlapping peak sets) were examined. **(B)** Distribution of ChIP-seq peaks from untreated Sko1-WT and Sko1-MT samples across indicated gene features. “Imm. Dnstrm.” refers to the 1000 bases immediately downstream of the 3ʹ UTR-encoding sequence. “WT & MT” refers to peaks shared between the Sko1-WT and Sko1-MT sets, whereas “WT only” and “MT only” refer to peaks that are unique to the respective peak sets. **(C)** Distribution of aggregate peak numbers around the nearest transcriptional start site (TSS) for “WT & MT” and “MT only” peak sets. **(D)** Percentage of peaks, within indicated peak sets, that contain the major Sko1 consensus motif, ATGACGT, or a less-specific motif, TKACG, where K is G or T.

We next performed a detailed analysis of the Sko1-WT and Sko1-MT peaks derived from normally growing yeast (untreated) from the overlapping peak sets. Nearly all the 207 Sko1-WT binding sites were also bound by Sko1-MT, but Sko1-MT was found at an additional 277 sites (Fig 4A). This is not the result of random binding of Sko1-MT at positions across the genome because ~90% of the peaks that are unique to Sko1-MT (“MT only”) are found near promoter regions, which is only slightly higher than the ratio of promoter-associated peaks in the Sko1-WT set (Fig 4B). Promoter regions include 2000 nt upstream, and 200 nt downstream, of transcriptional start sites (TSSs), which encompasses the upstream activation sequences (UASs) to which TFs typically bind [60]. Peaks that are unique to Sko1-WT (“WT only”) showed a somewhat different distribution, with about one third appearing in regions immediately downstream of gene ends, but this might be skewed by the small number of peaks (20), and its significance is not known (Fig 4B). For a more detailed analysis of the position of peaks, we plotted their distribution around TSSs (Figs 4C and S3A). MT-only peaks show a slightly more focused distribution than peaks that are common to both the Sko1-WT and MT sets (“WT & MT”), but they are predominantly situated around 400 to 500 bp upstream of TSSs, which is similar to the distribution of WT & MT peaks. These results indicate that Sko1 that cannot be sumoylated binds to numerous additional promoter regions in normally growing yeast, thereby implicating sumoylation in binding site specificity.

We then examined the nature of the Sko1-WT and Sko1-MT peaks to determine whether binding sites unique to Sko1-MT have common or distinguishing features. Genes situated nearest the Sko1-WT peaks are involved in diverse pathways, but Gene Ontology (GO) term analysis indicates that glucose, hexose, and ethanol metabolic processes are significantly enriched among these (*P* < 1.0 x 10^−4^), which matches the results of a previous examination of the Sko1 regulon [49]. GO term analysis for peaks unique to Sko1-MT, however, showed no GO term enrichment, indicating that there is no apparent bias in the distribution of MT-only binding sites with respect to target gene function.

To explore whether the SUMO-site mutation might alter the Sko1 binding sequence specificity, we determined the frequency at which the consensus Sko1 binding motif appears in Sko1-WT and Sko1-MT peak sets. About 60% of Sko1-WT peaks contain the sequence ATGACGT, whereas it is found in only ~12% of MT-only peaks (Fig 4D), strongly suggesting that blocking sumoylation alters Sko1 binding specificity. When we repeated this analysis with a less-specific version of the Sko1 binding site, TKACG (where K is G or T; based on the logo in Fig 3D), we found that this sequence is present in >80% of both Sko1-WT and MT-only peaks (Fig 4D). This implies that Sko1-MT recognizes Sko1 binding motif-like sequences, but with less stringency than Sko1-WT. In support of this, in a de novo motif discovery analysis, the only significant recurring motif identified in the MT-only peak set is a slightly weaker match to the consensus Sko1 motif, and it is found in a smaller fraction of peaks, when compared to the most recurring motif in the WT & MT set (S3B Fig). Taken together, our analysis suggests that sumoylation functions in preventing the association of Sko1 with non-specific binding sites that show some sequence similarity to its consensus binding motif.

### Sko1-MT binds more promoter regions than Sko1-WT during osmotic stress

**Fig 5:**
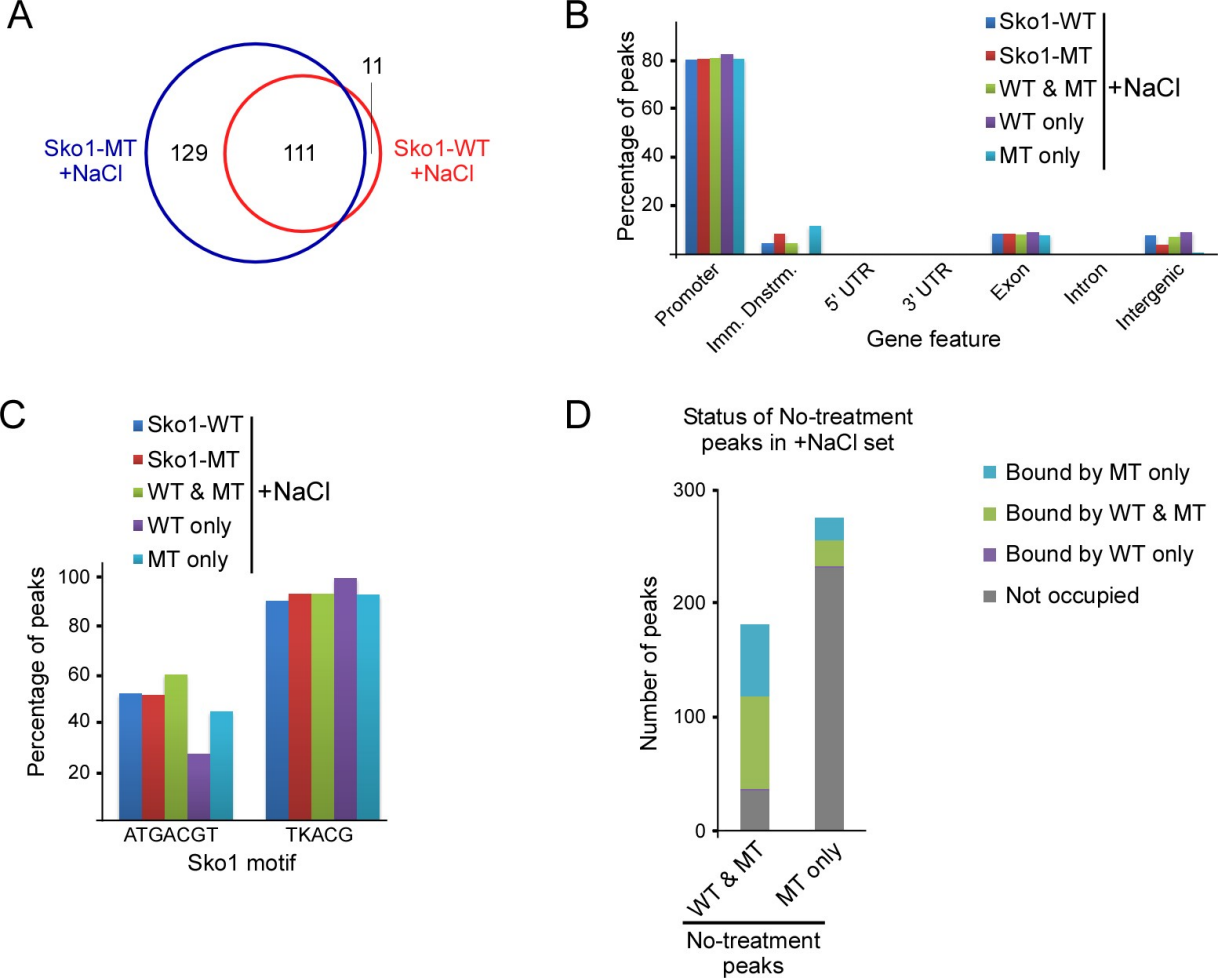
Sko1-MT binds more promoter regions than Sko1-WT after treatment with NaCl. **(A)** Venn diagram showing number of common and unique peaks from the NaCl-treated Sko1-WT and Sko1-MT ChIP-seq analysis. Peaks identified in both ChIP-seq replicates (overlapping peak sets) were examined. **(B)** Distribution of ChIP-seq peaks from NaCl-treated Sko1-WT and Sko1-MT samples across indicated gene features, as in Fig 4B. **(C)** Percentage of peaks, within indicated peak sets, that contain the major Sko1 consensus motif, ATGACGT, or a less-specific motif, TKACG, where K is G or T. **(D)** Status of “WT & MT” and “MT only” Sko1 binding sites from ChIP-seq analysis of untreated samples in the NaCl-treated set.

In both *SKO1-WT* and *sko1-MT* strains, osmotic stress resulted in a partial redistribution of Sko1 with many binding sites gained and several lost, which demonstrates that high osmolarity influences Sko1 binding site selection (Figs 3B, S2B and S2E). We analyzed the overlapping peak sets obtained by ChIP-seq after treatment with NaCl, which again indicated that Sko1-MT binds significantly more sites than Sko1-WT (240 peaks for MT versus 122 for WT; Fig 5A). Approximately 80% of all +NaCl Sko1-bound sites, including the 129 bound only by Sko1-MT and the 111 bound by both WT and MT, are near promoters (Fig 5B). De novo motif discovery indicates that the consensus Sko1 binding site is the most significantly recurring motif for both the WT & MT and MT-only +NaCl peak sets (S3B Fig), with the consensus motif appearing in ~45% of MT-only peaks and in ~60% of WT & MT peaks (Fig 5C). This is a greater fraction of MT-only peaks that contain the Sko1 binding site than in the untreated set (~45% vs ~12%; compare Figs 4D with 5C), which suggests that the effect of osmotic stress on Sko1 binding site selection outweighs the tendency for Sko1-MT to interact with degenerate binding sites. Consistent with this, high osmolarity likely influences Sko1 redistribution through phosphorylation of Sko1 by Hog1 [50], which we have shown occurs independently of its sumoylation (see above). Our results indicate that Sko1-MT binds many more sites than Sko1-WT in both untreated and +NaCl conditions, but binding appears to be more stringent for Sko1-MT under osmotic conditions, as more of the additional sites match the Sko1 consensus motif than under normal conditions.

To explore the effects of osmotic stress on Sko1 binding site redistribution, we examined the status of peaks from the untreated WT & MT and MT-only samples in the +NaCl peak sets (Fig 5D). About 75% of sites that were common to both Sko1-WT and Sko1-MT sets during normal growth remained bound during osmotic stress (Fig 5D). Strikingly, however, 83% of sites bound only by Sko1-MT during normal growth were unbound after treatment with NaCl. This indicates that Sko1-MT is dissociated from most of its nonspecific binding sites after exposure to NaCl, which suggests that binding to these sites can be less stable than to actual Sko1 binding sites. Altogether, our analysis supports the notion that sumoylation acts to restrict Sko1 to appropriate stable binding sites, even during osmotic stress.

### Sumoylation-deficient Sko1 shows higher occupancy levels at its binding sites

**Fig 6:**
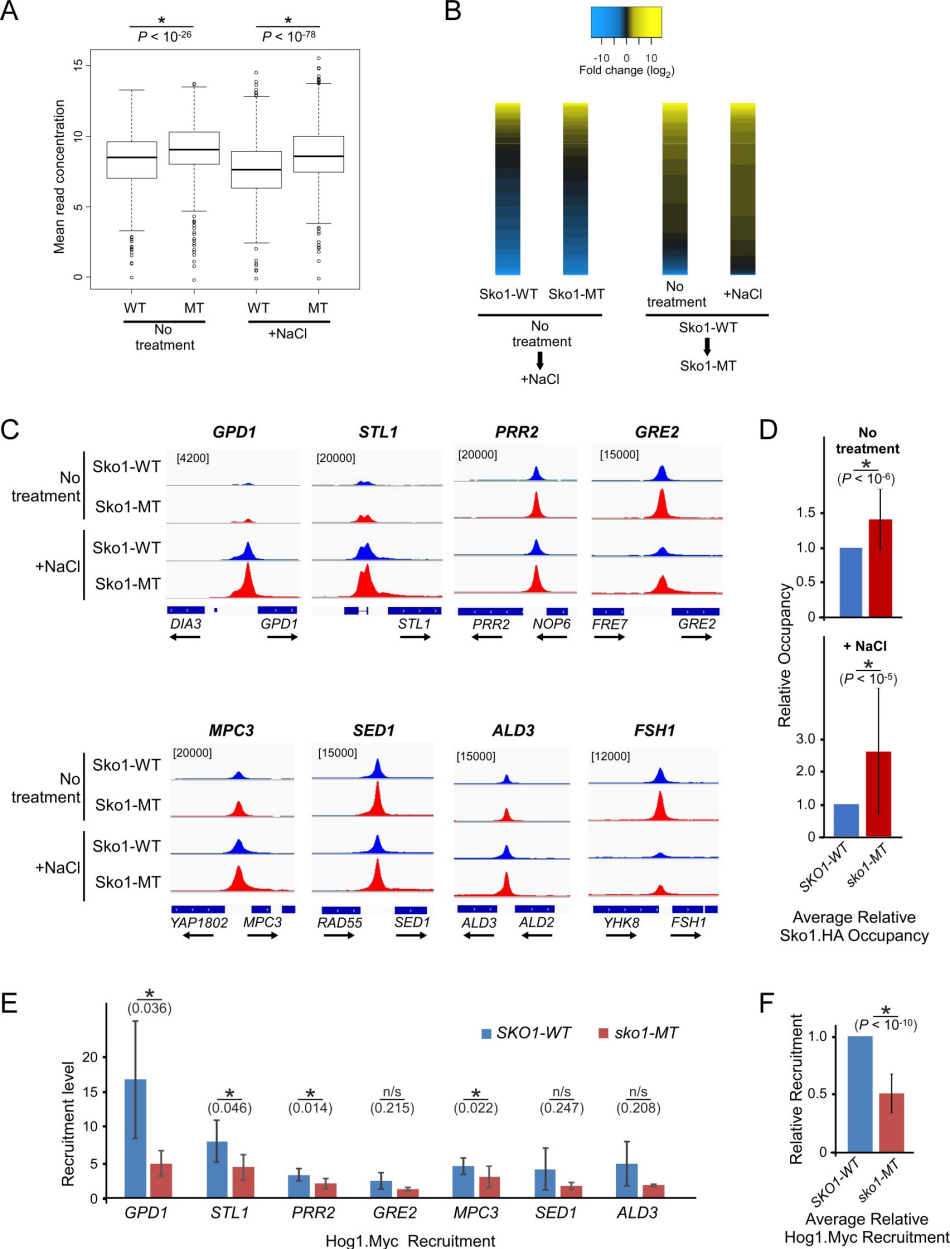
Differential binding analysis indicates that Sko1-MT has higher occupancy levels than Sko1-WT. **(A)** Boxplots comparing mean read concentrations (log_2_ normalized ChIP read counts) for the 630 consensus peaks in each of the four indicated ChIP-seq analyses. **(B)** At left, heatmap of differential binding analysis showing changes in occupancy levels (fold change; log_2_) in Sko1-WT or Sko1-MT after treatment with NaCl, for the 630 consensus peaks. Yellow indicates increased binding after NaCl, whereas blue indicates reduced binding. At right, heatmap of differential binding analysis comparing occupancy levels in Sko1-WT with Sko1-MT in untreated or NaCl-treated samples, at the 630 consensus peaks. Yellow indicates higher occupancy levels for Sko1-MT, whereas blue indicates higher occupancy for Sko1-WT. In all cases, black indicates no significant difference in occupancy level. Each heatmap is independently sorted by magnitude of occupancy level difference. **(C)** Sko1 occupancy peaks were aligned to the yeast genome and eight representative peak alignments are shown for the four samples analyzed from Replicate 1, as plotted using the Integrative Genomics Viewer (IGV). Numbers in square brackets refer to maximum data range (of sequence reads) for the tracks in each column. **(D)** Validation of ChIP-seq was performed at eight genes by qPCR analysis of five independent ChIP experiments (as summarized in S4B Fig). The average occupancy of Sko1-MT, relative to Sko1-WT occupancy, across all tested genes and replicates, is shown, in untreated samples (top), and in samples treated with 0.4 M NaCl for 5 min (bottom). Error bars represent standard deviation. **(E)** Recruitment level of Hog1.Myc, as determined by ChIP analysis (see S6 Fig), at indicated promoter regions in *SKO1-WT* and *sko1-MT* strains. Recruitment level was calculated as the ratio of occupancy at 5 min to occupancy at 0 min after treatment with NaCl. Error bars represent standard deviation of three independent replicates. **(F)** Average Hog1.Myc recruitment level in *sko1-MT* relative to its level in *SKO1-WT*. The level of Hog1.Myc recruitment in *sko1-MT*, calculated relative to recruitment in *SKO1-WT*, was averaged across all seven Hog1 target genes tested by ChIP-qPCR (panel *(E)* and S6 Fig). Error bars represent standard deviation for three replicates across all seven genes. *P*-values from Student’s *t*-test analysis are indicated. Asterisks (*) indicate that the compared data pairs are statistically different (*P* < 0.05; see *Materials and Methods*).

To investigate whether sumoylation can influence the occupancy level of Sko1, we next compared Sko1-WT and Sko1-MT occupancy levels at common binding sites across the genome. For this analysis, the DiffBind analysis tool was applied, which used a normalized number of sequence reads as a measure of occupancy across a consensus set of 630 peaks. The consensus peak set consists of peaks that were found in at least two of the four independent ChIP-seq analyses from both replicates, as listed in S5 Table. Significantly, as shown in the boxplots in Fig 6A and in S5 Table, Sko1-MT had overall higher occupancy levels than Sko1-WT in both untreated and +NaCl sets. To be sure that the effect is not only a result of the high number of MT-only peaks in the consensus peak set, two additional peak sets were also examined: (i) the set of 52 peaks that are present in both replicates of all four ChIP-seq analyses and, (ii) the 212 peaks that are present in both replicates of the Sko1-WT analyses in either the untreated or +NaCl sets, which can be considered normal Sko1 binding sites. In all cases, Sko1-MT showed significantly higher occupancy levels than Sko1-WT (S4A Fig).

To visualize the differences in occupancy levels at each of the 630 consensus peaks, heatmaps were generated (Fig 6B). For both Sko1-WT and Sko1-MT, osmotic stress resulted in an overall similar redistribution, with some sites showing increased binding (shown in yellow), but slightly more sites showing reduced binding (blue; Fig 6B; left pair of heatmaps). When comparing occupancy levels of Sko1 in *SKO1-WT* versus *sko1-MT* strains, however, most sites showed higher levels of Sko1-MT, and the effect was similar but more pronounced after treatment with NaCl (yellow; Fig 6B; right pair of heatmaps). Increased occupancy levels can also be seen in the unnormalized peak alignments shown in Fig 6C near eight selected representative Sko1 target genes, including those that show Sko1 recruitment (*GPD1*, *STL1*, *MPC3*, and *ALD3*), release of Sko1 (*GRE2*, *FSH1*), or no observable change in Sko1 occupancy in response to NaCl treatment (*PRR2*, *SED1*). To validate these results, we performed independent ChIP experiments followed by qPCR analysis of the promoter regions of the eight representative Sko1 target genes. Indeed, most genes showed statistically significantly higher levels of Sko1-MT compared with Sko1-WT in untreated and NaCl-treated samples (S4B Fig). When averaged across all eight genes, Sko1-WT occupancy was ~1.4 times greater than Sko1-MT for untreated samples, and ~2.6 times greater in the +NaCl samples (Fig 6D). Taken together, both our ChIP-seq and ChIP-qPCR experiments strongly indicate that sumoylation-deficient Sko1 shows increased occupancy at the majority of its binding sites and suggest that sumoylation decreases the binding affinity of Sko1.

### Blocking Sko1 sumoylation reduces Hog1 recruitment to target promoters

To explore the consequences of increased occupancy of Sko1-MT compared to Sko1-WT, we examined expression levels of some Sko1 target genes in *SKO1-WT* and *sko1-MT* cells, in both normal growth conditions and at various time points after treatment with NaCl. Blocking Sko1 sumoylation (*sko1-MT*) resulted in reduced RNA levels of one gene, *FSH1*, both prior to and after NaCl treatment, but had no significant effect on expression of the inducible genes *STL1*, *PRR2*, or *MPC3* (S5A Fig), suggesting that the consequences of Sko1 sumoylation on target gene expression are gene-specific. We also determined the mRNA levels of a selection of genes that were not bound by Sko1-WT but were bound by Sko1-MT in our ChIP-seq analyses. For all eight of these genes, there was no significant difference in expression level in the *SKO1-WT* and *sko1-MT* strains, both under normal conditions and 10 min after treatment with NaCl (S5B Fig). This indicates that binding of Sko1-MT alone is not sufficient to significantly alter the expression of genes that it does not normally regulate.

We then examined whether Sko1 sumoylation can influence the recruitment of Hog1, which associates with many Sko1 target genes during osmotic stress [47,50]. Strains expressing a 3xMyc-tagged version of Hog1 were generated in the *SKO1-WT* or *sko1-MT* backgrounds, and we performed Myc ChIP analysis over a NaCl-treatment time-course. The analysis showed robust but transient recruitment of Hog1 to seven of its known target genes in *SKO1-WT* cells after addition of NaCl, but not to *FSH1*, which is not regulated by Hog1 (S6 Fig). Two genes, *STL1* and *PRR2*, showed significantly reduced levels of Hog1 occupancy in the *sko1-MT* strain at 5 min after addition of NaCl. More significantly, however, in the *sko1-MT* strain, recruitment of Hog1, which we calculated as the ratio of occupancy at 5 min to 0 min post NaCl treatment, showed a statistically significant reduction at four of the seven genes (Figs 6E and S6). Indeed, when the average recruitment level was calculated across all seven genes, *sko1-MT* cells showed only 50% as much Hog1 recruitment as in *SKO1-WT* cells (Fig 6F). These observations suggest that sumoylation prevents excessive binding of Sko1 at target sites, and that elevated Sko1 binding affects expression of some genes and can significantly impair the recruitment of Hog1.

## Discussion

SUMO is a predominantly nuclear modifier that targets a large number of chromatin-associated proteins, including chromatin remodelers, general and sequence-specific transcription factors, and proteins involved in mRNA transport and processing [3,4,19,61]. Potentially then, the expression of numerous genes can be controlled by the cumulative effects of sumoylation of multiple substrates. Unlike ubiquitination, sumoylation in yeast and mammals involves a single E2 conjugating enzyme and a small number of E3 ligases and proteases [1]. This suggests that regulating the activity of only one enzyme in the SUMO pathway, such as Ubc9, can have widespread consequences for the cell since so many substrates may be coordinately affected. This scale of vast protein regulation, particularly on chromatin, might be necessary for achieving a specific goal, such as the dramatic refocusing of the transcription machinery to specific genes after exposure to heat stress [62]. However, unstressed cells show substantial levels of sumoylation and SUMO modification of numerous chromatin-associated proteins is constitutive, implying that this modification functions primarily in the mechanisms of gene expression under normal growth conditions [3,8]. Our analysis of Sko1 sumoylation supports this. Although osmotic stress resulted in the redistribution of Sko1 across the genome, its sumoylation level did not change, and blocking its sumoylation increased the number of its binding sites both in the presence or absence of NaCl stress.

Altogether, our data point to a role for SUMO modification in preventing Sko1 from binding to nontarget sites, genome-wide, which supports a novel function for the modification. Intriguingly, nonspecific sites bound by sumoylation-deficient Sko1 are not randomly distributed across the genome but are mostly situated near gene promoters that contain Sko1 binding site-like sequences. This might reflect the intrinsic accessibility of chromatin around promoters and the frequent occurrence of such sequences in many promoter regions [63]. Indeed, binding motifs for Sko1 and the related TFs Aca1 and Cst6 are highly similar, and it has been proposed that these TFs compete with each other for binding to promoter elements [40,45,64]. Based on the results of our study, we propose a model in which unbound Sko1 has high affinity for sequences that generally resemble the Sko1 motif. This might be necessary to ensure that all functional Sko1 target sites become occupied by the TF, even though nonspecific sites are initially bound, as well. Our model then posits that subsequent sumoylation of Sko1 relaxes its interaction with bound DNA, resulting in release from inoptimally bound nonspecific sites but allows sustained association with actual Sko1-regulated genes. Key to this model is that sumoylation takes place only after Sko1 has bound DNA, which is supported by our finding that DNA binding mutations eliminate Sko1 sumoylation. Similarly, DNA binding mutations have been shown to prevent sumoylation of other TFs such as Gcn4 and Ikaros, as well as non-TF DNA-binding proteins, including yeast Yku70, human TDG and viral ULFF, which are involved in DNA damage repair [28,65,66]. This suggests that the sumoylation apparatus can distinguish between DNA-bound and unbound forms of many chromatin-associated proteins, possibly as a result of conformational changes that may occur after binding DNA, or due to the proximity of the sumoylation machinery with chromatin. Supporting this, ChIP analyses have shown that Ubc9 is associated with chromatin, including promoter regions of transcriptionally activated genes [14,15]. Further studies are necessary to test our model and explore the consequences of Sko1 sumoylation on its structure and DNA binding affinity.

Consistent with the idea that sumoylation reduces Sko1 DNA binding affinity, blocking Sko1 sumoylation also resulted in increased occupancy levels at most of its target genes. However, not all target genes showed altered expression in the *sko1-MT* strain. The increase in Sko1 levels at these sites might not have been sufficiently dramatic to noticeably alter their gene expression levels, but this supports the previous finding that Sko1 occupancy levels on target genes generally do not correlate with their expression levels [49]. Instead, its context-dependent roles involve regulating the recruitment or release of repressors, such as Tup1/Ssn6, chromatin remodelers SAGA and SWI/SNF, and the kinase Hog1 [44,47,50]. Hog1 is activated in response to osmotic stress which triggers its nuclear localization and association with promoters of osmo-regulated genes where it phosphorylates a number of transcription-related substrates, including Sko1 and RNAP II, resulting in efficient induction of the genes [67,68]. Whereas Sko1 itself is required for the recruitment of Hog1 to at least some target genes during osmotic stress [47,50], our results suggest that increased binding of Sko1 in *sko1-mt* cells generally hinders the recruitment of Hog1 to its targets. Increased Sko1 binding in these cells might inhibit promoter-associated rearrangements that are necessary for induction of osmoregulated genes [47], or Sko1 sumoylation itself might stimulate the recruitment of Hog1 through unknown mechanisms. In any case, our results point to a role for Sko1 sumoylation in controlling its association with chromatin not only to ensure binding site specificity, but also to prevent excessive binding at its authentic target sites.

Few studies to date have examined the role of sumoylation on the genome-wide occupancy TFs, and, when considered with our results, the conclusions of these studies strongly point to a conserved role for sumoylation in regulating TF binding specificity. In the first such study, it was reported that a germline mutation in the human TF MITF that is associated with increased coincidence of melanoma and renal cancer also significantly reduces sumoylation of the protein [30]. ChIP-seq analysis was performed in human melanoma cell lines expressing either WT MITF or the sumoylation-deficient form, MITF-E318K. As in our analysis with Sko1, dramatically more sites across the genome were bound by the sumoylation-deficient form of MITF, and occupancy levels were higher compared with WT MITF at sites that were bound by both the mutant and WT proteins. Recent ChIP-seq studies of the glucocorticoid and androgen nuclear receptors again showed that SUMO-site mutation led to altered genome occupancy patterns for both TFs, with the mutant proteins binding to significantly more sites than the wild-type counterparts [32,33]. The parallel observations of these analyses of different TFs in evolutionarily distant organisms strongly suggests a common function for sumoylation across eukaryotes. Considering the vast number of eukaryotic TFs that have been reported as SUMO targets and the close association of the modification with chromatin, we anticipate that future additional genome-wide studies will reveal that, indeed, a major role for sumoylation is in regulating the association of DNA-bound TFs with chromatin in order to restrict their activity to appropriate target genes.

## Materials and methods

### Yeast strains and plasmid

Yeast strains used in this study are listed in S1 Table. Sko1- and Hog1-tagged strains were epitope tagged using homologous recombination as previously described [69]. Strains with a *SKO1* deletion were generated using a KanMX deletion cassette by homologous recombination. Plasmid Gal4DB-K450E was generated by PCR-based sub-cloning of the *sko1-K450E* coding sequence into vector pGBT9 at the SmaI restriction site. It was then transformed into the HF7c yeast strain, which contains two genomic reporter genes with Gal4 binding sites.

### Yeast growth assay (“spot assay”)

Yeast cultures were grown overnight in appropriate liquid medium and diluted to an optical density (OD at 595 nm) of 0.2. Then, cells of each strain were spotted side-by-side in five-fold serial dilutions on solid-media plates with or without indicated osmotic stress conditions. Plates were incubated at 30°C and images were taken after the indicated durations.

### Preparation of yeast lysates and immunoprecipitation (IP)

Yeast cultures (40–50 mL) were grown in appropriate liquid medium to mid-log phase (OD_595 nm_ of 0.5 to 0.7). Osmotic stress was induced by adding NaCl to a final concentration of 0.4 M for the indicated time points. Cells were then harvested by centrifugation at 3000 *g* for 5 min, followed by a wash with IP buffer (50 mM Tris-HCl, pH 8, 150 mM NaCl, 0.1% Nonidet P-40 (NP40), 1X yeast protease inhibitor cocktail (BioShop), 1 mM phenylmethylsulfonyl fluoride (PMSF), and 2.5 mg/mL *N*-ethylmaleimide (NEM)). Cells were lysed with glass beads in IP buffer containing 0.1 mM dithiothreitol for 30 min at 4˚C. The lysed material was isolated from the beads and centrifuged twice to remove insoluble materials. Samples were either analyzed by immunoblot or used for IP. For IP experiments, an aliquot of yeast lysate was retained as input sample, and the remainder was incubated overnight with anti-HA agarose beads or Protein G agarose with HA antibody at 4˚C. IPs were washed three times with ice-cold IP buffer plus 0.1% NP40, and twice with IP buffer alone. Beads were then resuspended and boiled with SDS loading buffer for 3 min, prior to analysis by the indicated immunoblots.

### Denatured immunoprecipitation (IP)

Yeast cultures (50 mL) were grown in YPD medium to an OD_595 nm_ of ~0.65. Cells were harvested by centrifugation at 3000 *g* for 5 min and washed with 20% trichloroacetic acid (TCA). Washed cells were then lysed with glass beads in 20% TCA. Lysates were precipitated and washed with 5% TCA and resuspended with modified SDS buffer (60 mM Tris pH 6.7, 5% 2-mercaptoethanol, 1% SDS, few drops of a bromophenol blue solution) prior to boiling for 5 min. Boiled samples were centrifuged at room temperature for 10 min. An aliquot (40 μL) of the supernatant was retained as input sample and the remainder was diluted with denaturing IP buffer (50 mM Tris pH 7.4, 150 mM NaCl, 0.5% NP40) containing 0.5 mg/mL of bovine serum albumin. Diluted samples were then incubated overnight with anti-HA agarose beads at 4˚C. IPs were washed three times with ice-cold denaturing IP buffer. Beads were then resuspended and boiled with SDS loading buffer for 3 min prior to analysis by the indicated immunoblots.

### Chromatin immunoprecipitation (ChIP)

Yeast cultures (50 mL) were grown in YPD medium and induced by osmotic stress as indicated for IP procedure. Cells were then cross-linked with 1% formaldehyde for 20 min, followed by 5 min of quenching with 282 mM of glycine. Cells were pelleted by centrifugation and washed twice with ice-cold TBS (20 mM Tris-HCl, pH 7.5 and 150 mM NaCl), then in ChIP buffer (50 mM HEPES-KOH, pH 7.5, 150 mM NaCl, 1 mM EDTA, 1% Triton X-100, 0.1% sodium deoxycholate and 0.1% SDS). Washed samples were resuspended in ChIP buffer and lysed with glass beads using a mini bead beater. The lysed materials were isolated from glass beads and sonicated to yield an average DNA fragment size of 300 to 500 bp in length. Samples were then centrifuged at 14,000 *g* for 5 min. To the isolated supernatants, additional NaCl was added to a final concentration of 225 mM. For IP, salt-adjusted supernatants were incubated with 15 μL magnetic Protein G beads (Dynabeads, Thermo Fisher Scientific) pre-bound with 2 μg rabbit anti-HA (Novus) or mouse anti-MYC (NEB) antibodies. IPs were washed with four different buffers for 4 min in the following order: (1) ChIP buffer with 275 mM NaCl; (2) ChIP buffer with 400 mM NaCl; (3) A buffer containing 10 mM Tris-HCl, pH 8, 0.25 M LiCl, 1 mM EDTA, 0.5% NP-40 and 0.5% sodium deoxycholate; (4) Tris-EDTA buffer (10 mM Tris-HCl, pH 8 and 1 mM EDTA). Washed beads were then incubated in ChIP elution buffer (50 mM Tris-HCl, pH 7.5, 10 mM EDTA and 1% SDS) for 10 min at 65°C. Bound material was isolated on a magnet, treated with RNase for 30 min at 37°C, then with proteinase K at 42°C for 1 h. To reverse crosslinking, samples were incubated overnight at 65°C. DNA was purified and recovered using GeneJet Gel Extraction Kit (Thermo Fisher) followed by a quantitative PCR using primers listed in S2 Table. Results from qPCR were normalized to an untranscribed region of Chromosome V for Fig 2C, then an internal control gene, *PMA1*, for other ChIP analyses. ChIP experiments were repeated at least three times and the averages were plotted with the standard deviations shown as error bars. Pair-wise statistical analyses were performed by Student’s *t*-test (Figs 2C, 6A, 6D, 6E, 6F and S6), whereas statistical comparison of sets of data in S4B and S5 Figs were performed by ANOVA. Asterisks indicate a significant difference with *P*-values less than 0.05 for ChIP and RT qPCR analyses, whereas pairs and sets with *P*-values greater than 0.05 are unmarked or marked as “n/s.”

### Isolation of RNA and reverse transcription (RT)

RNA was isolated as previously reported from 10 mL yeast culture and subjected to DNase treatment [70]. Reverse transcription was performed on 1 μg of DNA-free-RNA using iScript cDNA synthesis (Bio-Rad) followed by qPCR. mRNA levels were normalized to an internal loading control, 25S rRNA. Primer sequences used for these experiments are listed in S2 Table. Error bar represents the standard deviation of three replicates.

### ChIP-seq and analysis

For ChIP-seq analysis, ChIP was performed as described above, but scaled up as appropriate. Namely, culture volumes were 200 mL in rich (YPD) medium, and 6 μg of HA antibody was pre-bound to 40 μL of Dynabeads. Two replicates of the ChIP-seq experiment described above were performed, with sequencing libraries generated for both IP and input samples for Replicate 1, but only IP samples were sequenced for Replicate 2. Libraries were prepared using the NEBNext Ultra II DNA library prep kit (New England Biolabs), and paired-end sequencing (two times 126 bases) was performed using an Illumina HiSeq 2500 instrument at The Centre for Applied Genomics (TACG) at the Hospital for Sick Children (Toronto). Raw and processed sequencing data files have been uploaded to the NCBI Gene Expression Omnibus (GEO) with accession number GSE118655.

For differential binding analysis, which was performed at TACG, sequencing reads were aligned, and peaks assigned as follows. Sequencing adaptors were trimmed using Trim Galore! (version 0.0.4) running Cutadapt (version 1.10) with the following parameters: quality score cut-off of 25, six nucleotides were removed from 5′ ends, Illumina universal adapter sequences were removed, stringency setting of 5, sequences shorter than 40 nt after trimming were discarded, and only pairs of reads were retained. Trimmed forward and reverse reads were then aligned to the sacCer3 reference genome using Bowtie2 (version 2.3.2) [71]. Peak calling was performed using the MACS2 peak assignment tool (version 2.1.1) [72] in paired-end mode, with a genome size of 1.2e7, and using corresponding input samples derived from Replicate 1 as controls for peak calling for both Replicates 1 and 2. The default statistical significance cut-off was applied: 0.05 for the *q*-value, which is the false-discovery rate (FDR)-adjusted *p*-value, calculated using the Benjamini-Hochberg correction. Fold enrichment (FE) is calculated as the fold enrichment for each peak summit against a random Poisson distribution with local lambda. A set of consensus peaks was assembled using DiffBind (version 2.2.12), such that each peak was identified in at least two independent samples from both replicates, or as otherwise noted. Binding affinities were determined and compared using DiffBind based on the number of ChIP read counts (log_2_ of normalized ChIP read counts with input read counts subtracted) (S5 Table). Because input samples were derived only for Replicate 1, and Replicate 2 had consistently lower read counts, the replicate number was used as a blocking factor for batch correction in the statistical model used by DiffBind when calculating the affinity scores. Peaks were annotated with all genomic features within 5 kb of consensus peaks using ChIPpeakAnno (version 3.12.7; Bioconductor Package) [73], with the reference genome annotation package TxDb.Scerevisiae.UCSC.sacCer3.sgdGene. Differential binding data provided by TACG was then used to generate heatmaps to display pair-wise fold changes in binding occupancy levels (Fig 6B) using the Heatmapper web tool [74].

For peak number analysis (Figs 3, 4, 5 and S2), sequence read alignments and peak calling were performed as described above, with the following notes and exceptions. Peak assignment normalization for Replicate 2 was performed using either the input controls from Replicate 1, or using local genomic bias from the ChIP samples, themselves (i.e. without a control) [72]. Both methods produced similar results, with the latter used to produce the data shown in Figs 4, 5 and S2. Some computations were performed using the Niagara supercomputer at the SciNet HPC Consortium. Lists of identified peaks, excluding peaks found with the mitochondrial genome, are presented in S3 Table. Peak analysis, including identifying overlapping peaks among data sets (findOverlapsOfPeaks function; S4 Table) and the distribution of peaks across gene features (assignChromosomeRegion function) were performed using ChIPpeakAnno. Frequency of motif occurrences within peak sets (Figs 4D and 5C) was determined using the summarizePatternInPeaks function within the ChIPpeakAnno toolset. De novo motif discovery was performed using the MEME analysis tool for indicated peak sets using 41 nt surrounding peak summits and restricted to motifs of 6 to 12 nt in length [75]. An analysis with 81 nt surrounding peak summits was also performed, which resulted in similar results. Visualization of peak alignments (Fig 6C) was performed using the Integrative Genomics Viewer (Broad Institute) [76].

## Supporting information

S1 Fig **(A) C-terminal 6xHA tag on Sko1 does not affect cell growth in normal or osmotic conditions.** Spot assays (as in Fig 1F) in which indicated yeast strains were grown in triplicate on rich (YPD) or synthetic complete (SC) medium, or supplemented with indicated levels of NaCl or sorbitol. Plates were photographed after two or three days, as indicated. **(B) Detection of Sko1 sumoylation in lysates prepared under denaturing conditions.** IP-immunoblot analysis was performed with protein samples prepared under denaturing conditions (TCA precipitation). Asterisks (*) indicate position of the major (mono-) sumoylated form of Sko1 in each immunoblot. Open circles (○) indicate position of putative Sko1 degradation products, detectable in both HA and SUMO immunoblots. (**C) A yeast strain expressing sumoylation-deficient Sko1 shows no growth defect.** Spot assays in which growth of indicated yeast strains were compared on rich (YPD) or synthetic complete (SC) medium, as in Fig 1F. Growth was for two days (2d). (**D) Sko1 sumoylation levels are unaffected by stress.** Relative Sko1 sumoylation levels were quantified after IP-immunoblot analyses as in Fig 1E by dividing Sko1 SUMO signals by the Sko1-HA signals in the respective blots. Data is presented relative to the untreated sample, with error bars indicating standard deviation of three experiments. By Student’s *t*-test, there is no significant statistical difference among the samples. (**E) Blocking Sko1 sumoylation does not affect its abundance.** HA and GAPDH immunoblot analysis of lysates from *SKO1*.*-WT*.*HA* or *sko1-MT*.*HA* strains grown in SC medium treated with 0.4 M NaCl for indicated times. Sumoylated forms of Sko1.WT cannot be seen in this short exposure. **(F) Blocking Sko1 sumoylation does not prevent its Hog1-mediated phosphorylation.** HA immunoblot analysis, as in Fig 2B, using Phos-Tag acrylamide to enhance detection of phosphorylated forms of Sko1.HA, indicated as “Sko1-P.” A strain lacking *HOG1* and expressing Sko1.HA was included as a control. Analysis using standard SDS-PAGE analysis is shown at bottom.
(PDF)

S2 Fig Binding site analysis of Sko1-WT and Sko1-MT ChIP-seq experiment for Replicate 2 and for peaks overlapping in both replicates. **(A)** Number of binding sites (peaks) identified from Replicate 2 ChIP-seq analysis of *SKO1-WT*.*HA* and *sko1-MT*.*HA* strains, either untreated or treated with 0.4 M NaCl for 5 min, with a *q-*value less than 0.05 (blue bars). Subset of peaks having a *q*-value less than 0.05 and a fold enrichment (FE) value greater than 2 are also indicated (red bars). **(B)** Venn diagram, as in Fig 3B, showing number of peaks (*q* < 0.05) shared among the four samples in Replicate 2. **(C)** Venn diagrams indicating numbers of peaks identified in both Replicate 1 and 2, for each of the four samples. Peaks found in both replicates (i.e. intersects) for each sample constitute the “Overlapping Peak Sets.” At right, similar analysis comparing peaks from Replicate 1 and the subset of peaks from Replicate 2 that have an FE greater than 2. All analyzed peaks have *q*-values less than 0.05. **(D)** Number of binding sites for each of four samples in the overlapping peak sets, which includes only peaks identified in both replicates (as indicated in *(C) left*). **(E)** Venn diagram showing number of common and unique peaks in the overlapping peak sets from the four samples.
(PDF)

S3 Fig Distribution and motif analysis of ChIP-seq peak sets. **(A)** Distribution of aggregate ChIP-seq peak numbers (for overlapping peak sets) around the nearest transcriptional start site (TSS) for indicated peak sets in untreated and NaCl-treated samples. **(B)** De novo motif discovery was performed, using the MEME analysis tool, for “WT & MT” and “MT only” peak sets, in untreated and NaCl-treated samples. Only one significant motif was identified for each of the untreated and +NaCl MT-only peak sets (with an *E*-value less than 1e-005), and the most significant motifs for the WT & MT peak sets are shown (additional motifs discovered for the WT & MT set have *E*-values greater than 1e-32 and are present in fewer than 40 peaks). Number of peaks contributing to the motif (“Sites”) is indicated in each case.
(PDF)

S4 Fig **(A) Differential binding analysis of different groups of peaks.** Boxplots comparing mean read concentrations (log_2_ normalized ChIP read counts) in each of the four indicated ChIP-seq analyses for the 52 peaks that are found in both replicates of all four ChIP-seq sets (left) or the 212 peaks that are found in both replicates of the Sko1-WT sets in both untreated and +NaCl conditions (right). **(B) Validation of ChIP-seq analysis.** Five independent standard ChIP experiments were performed with *SKO1-WT* and *sko1-MT* strains. Sko1.HA occupancy levels at promoter regions of eight representative genes were determined by qPCR, at 0 or 5 min after the addition of 0.4 M NaCl. For each gene, occupancy is shown relative to Sko1-WT in untreated samples. Error bars represent standard deviations. *P-*values from two-factor ANOVA analysis of WT vs MT sets for each gene are shown. Asterisks (*) indicate that the two data sets (WT and MT) are statistically different (*P* < 0.05; see Materials and Methods).
(PDF)

S5 Fig Effects of elevated Sko1 binding on steady-state expression levels of target genes in the *sko1-MT* strain. **(A)** Quantitative RT-PCR analysis of mRNA levels of indicated representative Sko1-target genes at 0, 10, 20 and 30 min after treatment of *SKO1-WT* or *sko1-MT* cultures with 0.4 M NaCl. Error bars represent standard deviations of three independent replicates. *P-*values from two-factor ANOVA analysis of WT vs MT sets for each gene are shown. Asterisks (*) indicate that the two data sets (WT and MT) are statistically different (*P* < 0.05; see Materials and Methods). **(B)** Quantitative RT-PCR analysis of mRNA levels of a selection of genes that are bound by Sko1-MT, but not Sko1-WT, at 0 and 10 min after treatment of *SKO1-WT* or *sko1-MT* strains with 0.4 M NaCl. Statistical analysis indicates no significant difference between WT and MT sets. Error bars represent standard deviations of four independent replicates.
(PDF)

S6 Fig Effects of blocking Sko1 sumoylation on recruitment of Hog1 to target genes during osmotic stress. ChIP-qPCR analysis of Hog1.Myc occupancy at indicated genes in *SKO1-WT* and *sko1-MT* strains at 0, 5, or 15 min after treatment with NaCl. Data are represented as fold occupancy (relative to occupancy at the *PMA1* locus which is not targeted by Hog1 or Sko1). Error bars represent standard deviations of three independent replicates. Asterisks (*) indicate that the compared data pairs are statistically different (*P* < 0.05; see Materials and Methods). Statistical comparison of Hog1.Myc recruitment is shown in Fig 6E.
(PDF)

S1 Table List of yeast strains used in this study. (DOCX)

S2 Table List of oligonucleotide sequences used in this study. (DOCX)

S3 Table List of peaks identified in ChIP-seq peak analysis for each of the four samples over two replicates (Replicates 1 and 2). (XLSX)

S4 Table Annotated list of peaks that are present in both replicates for each sample/analysis (“Overlapping Peak Sets”). (XLSX)

S5 Table Differential binding analysis (performed with DiffBind) at consensus peaks for pair-wise comparisons of ChIP-seq data sets. Data relates to Fig 6.
(XLSX)

S6 Table Numerical values for graphical data presented throughout, listed by figure number as separate sheets/tabs. (XLSX)
