# Supplementary figures and images for: Sumoylation of DNA-bound transcription factor Sko1 prevents its association with nontarget promoters
Source: PLoS Genet. 2019 Feb 14;15(2):e1007991. doi: 10.1371/journal.pgen.1007991 (PMC6392331; doi:10.1371/journal.pgen.1007991)

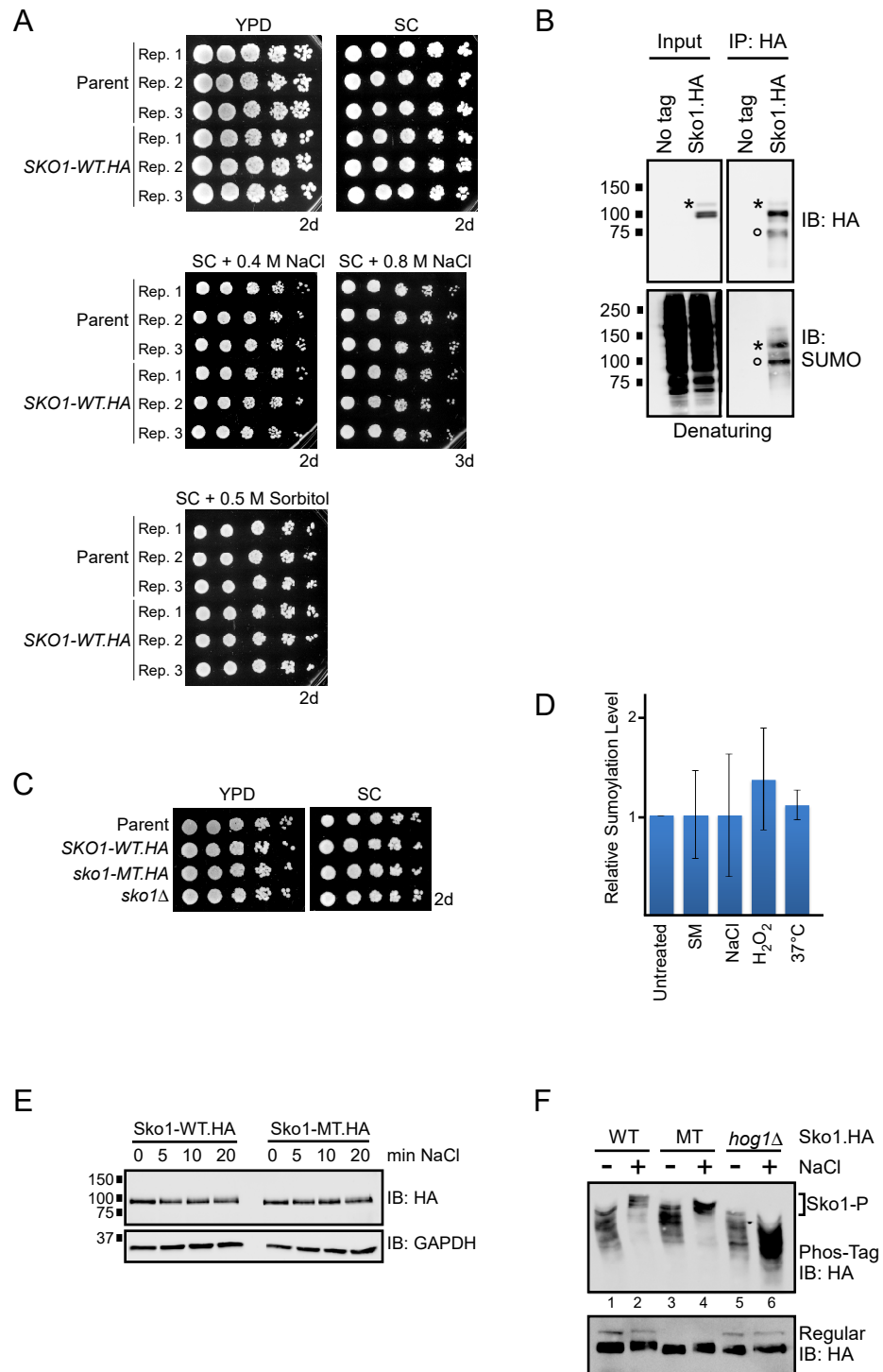

Supplement: S1 Fig — (A) C-terminal 6xHA tag on Sko1 does not affect cell growth in normal or osmotic conditions. Spot assays (as in Fig 1F) in which indicated yeast strains were grown in triplicate on rich (YPD) or synthetic complete (SC) medium, or supplemented with indicated levels of NaCl or sorbitol. Plates were photographed after two or three days, as indicated. (B) Detection of Sko1 sumoylation in lysates prepared under denaturing conditions. IP-immunoblot analysis was performed with protein samples prepared under denaturing conditions (TCA precipitation). Asterisks (*) indicate position of the major (mono-) sumoylated form of Sko1 in each immunoblot. Open circles (○) indicate position of putative Sko1 degradation products, detectable in both HA and SUMO immunoblots. (C) A yeast strain expressing sumoylation-deficient Sko1 shows no growth defect. Spot assays in which growth of indicated yeast strains were compared on rich (YPD) or synthetic complete (SC) medium, as in Fig 1F. Growth was for two days (2d). (D) Sko1 sumoylation levels are unaffected by stress. Relative Sko1 sumoylation levels were quantified after IP-immunoblot analyses as in Fig 1E by dividing Sko1 SUMO signals by the Sko1-HA signals in the respective blots. Data is presented relative to the untreated sample, with error bars indicating standard deviation of three experiments. By Student’s t-test, there is no significant statistical difference among the samples. (E) Blocking Sko1 sumoylation does not affect its abundance. HA and GAPDH immunoblot analysis of lysates from SKO1.-WT.HA or sko1-MT.HA strains grown in SC medium treated with 0.4 M NaCl for indicated times. Sumoylated forms of Sko1.WT cannot be seen in this short exposure. (F) Blocking Sko1 sumoylation does not prevent its Hog1-mediated phosphorylation. HA immunoblot analysis, as in Fig 2B, using Phos-Tag acrylamide to enhance detection of phosphorylated forms of Sko1.HA, indicated as “Sko1-P.” A strain lacking HOG1 and expressing Sko1.HA was included as a [file pgen.1007991.s001.pdf]

Supplementary Figure S2

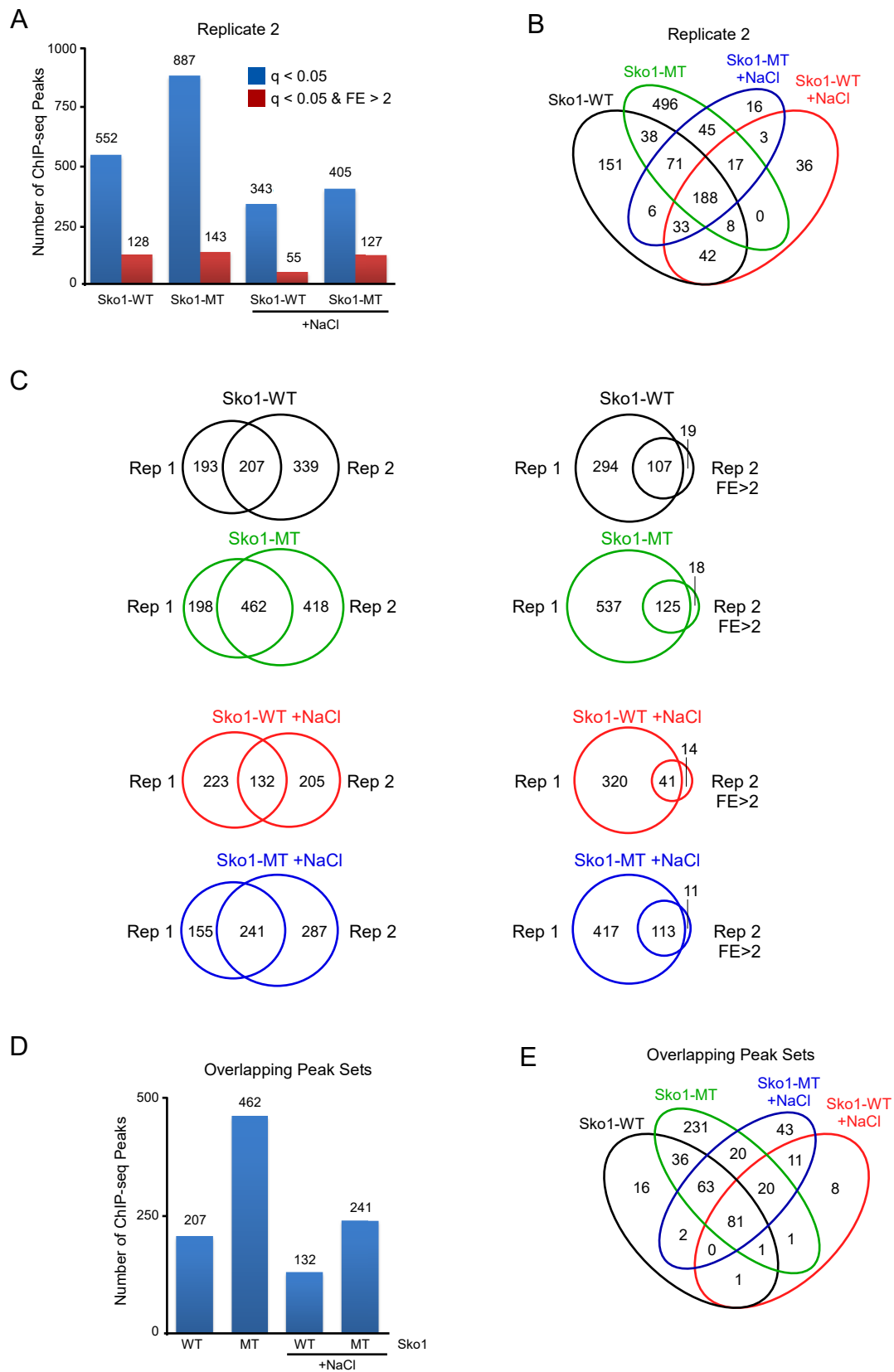

Supplement: S2 Fig — (A) Number of binding sites (peaks) identified from Replicate 2 ChIP-seq analysis of SKO1-WT.HA and sko1-MT.HA strains, either untreated or treated with 0.4 M NaCl for 5 min, with a q-value less than 0.05 (blue bars). Subset of peaks having a q-value less than 0.05 and a fold enrichment (FE) value greater than 2 are also indicated (red bars). (B) Venn diagram, as in Fig 3B, showing number of peaks (q < 0.05) shared among the four samples in Replicate 2. (C) Venn diagrams indicating numbers of peaks identified in both Replicate 1 and 2, for each of the four samples. Peaks found in both replicates (i.e. intersects) for each sample constitute the “Overlapping Peak Sets.” At right, similar analysis comparing peaks from Replicate 1 and the subset of peaks from Replicate 2 that have an FE greater than 2. All analyzed peaks have q-values less than 0.05. (D) Number of binding sites for each of four samples in the overlapping peak sets, which includes only peaks identified in both replicates (as indicated in (C) left). (E) Venn diagram showing number of common and unique peaks in the overlapping peak sets from the four samples. (PDF) [file pgen.1007991.s002.pdf]

A

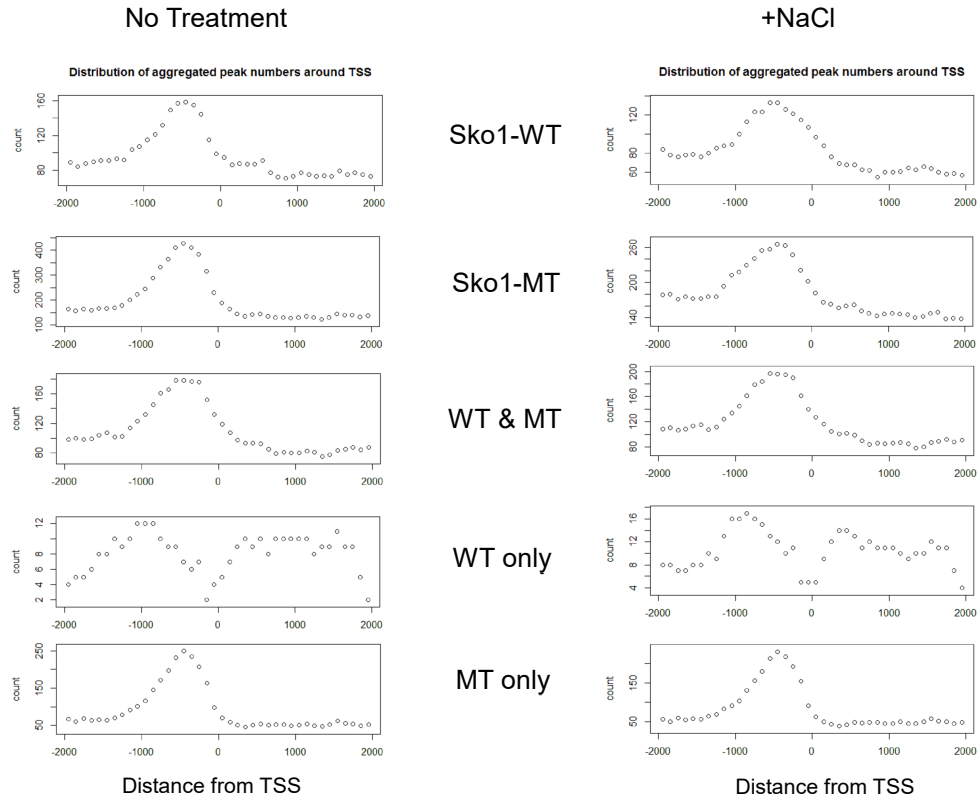

B

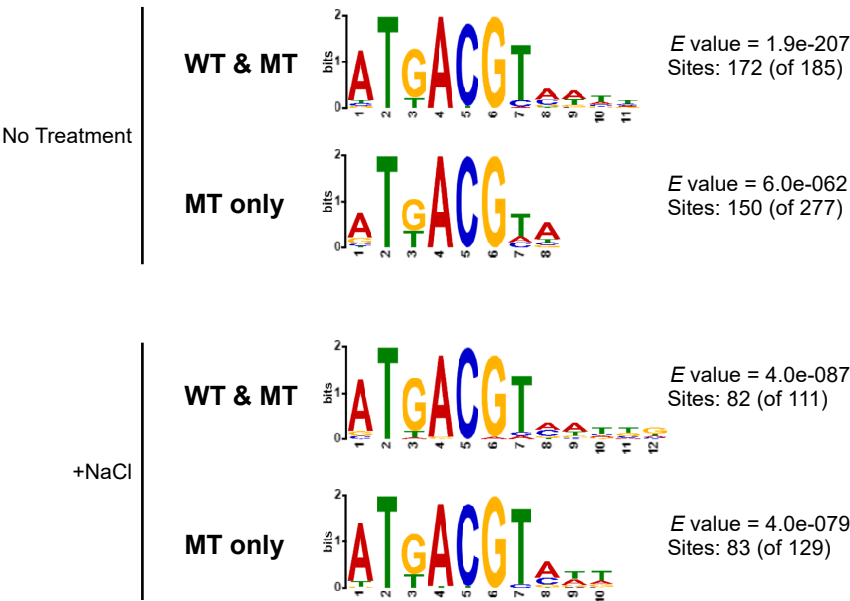

Supplement: S3 Fig — (A) Distribution of aggregate ChIP-seq peak numbers (for overlapping peak sets) around the nearest transcriptional start site (TSS) for indicated peak sets in untreated and NaCl-treated samples. (B) De novo motif discovery was performed, using the MEME analysis tool, for “WT & MT” and “MT only” peak sets, in untreated and NaCl-treated samples. Only one significant motif was identified for each of the untreated and +NaCl MT-only peak sets (with an E-value less than 1e-005), and the most significant motifs for the WT & MT peak sets are shown (additional motifs discovered for the WT & MT set have E-values greater than 1e-32 and are present in fewer than 40 peaks). Number of peaks contributing to the motif (“Sites”) is indicated in each case. (PDF) [file pgen.1007991.s003.pdf]

Supplementary Figure S5

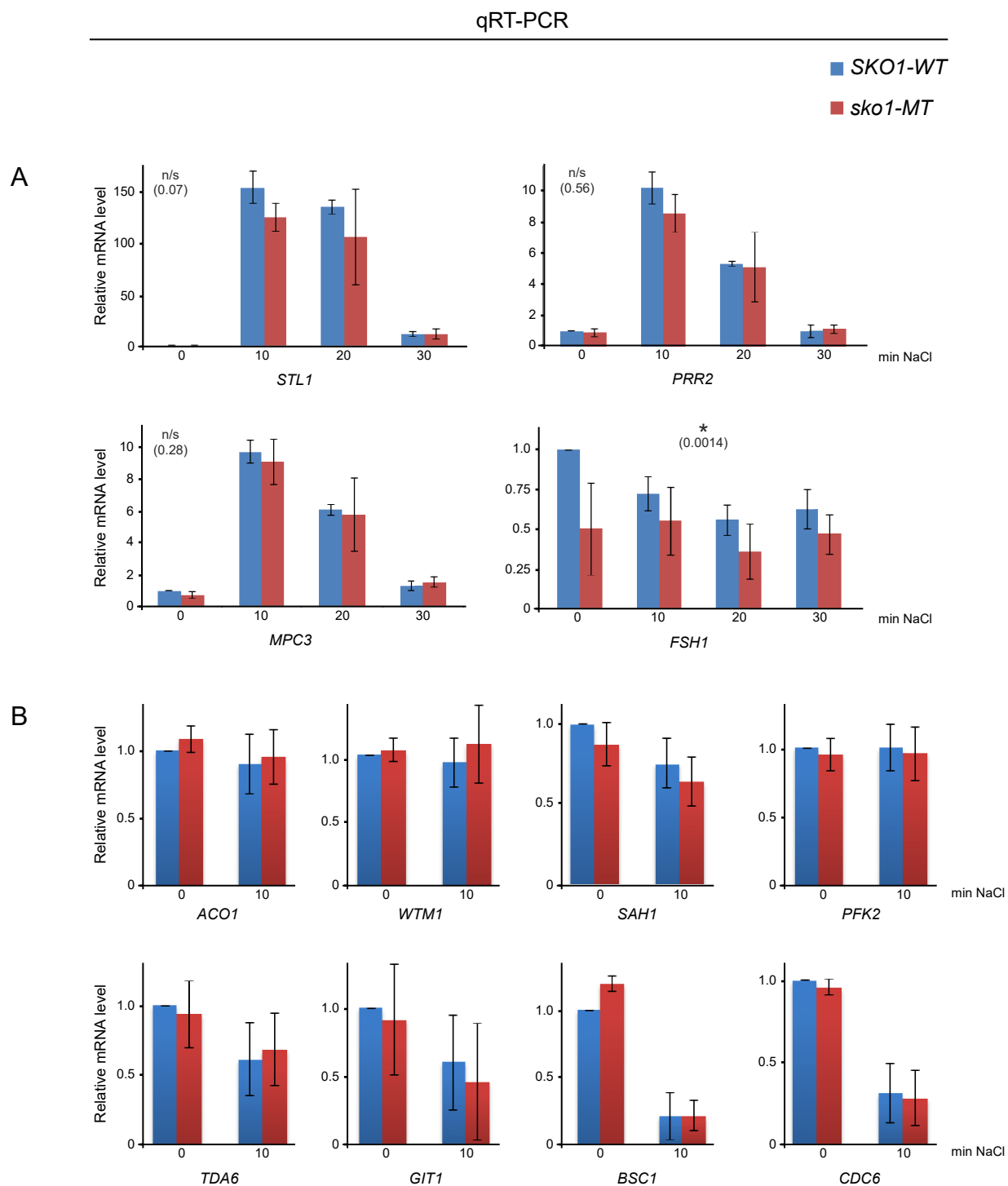

Supplement: S5 Fig — (A) Quantitative RT-PCR analysis of mRNA levels of indicated representative Sko1-target genes at 0, 10, 20 and 30 min after treatment of SKO1-WT or sko1-MT cultures with 0.4 M NaCl. Error bars represent standard deviations of three independent replicates. P-values from two-factor ANOVA analysis of WT vs MT sets for each gene are shown. Asterisks (*) indicate that the two data sets (WT and MT) are statistically different (P < 0.05; see Materials and Methods). (B) Quantitative RT-PCR analysis of mRNA levels of a selection of genes that are bound by Sko1-MT, but not Sko1-WT, at 0 and 10 min after treatment of SKO1-WT or sko1-MT strains with 0.4 M NaCl. Statistical analysis indicates no significant difference between WT and MT sets. Error bars represent standard deviations of four independent replicates. (PDF) [file pgen.1007991.s005.pdf]

## Hog1.Myc ChIP

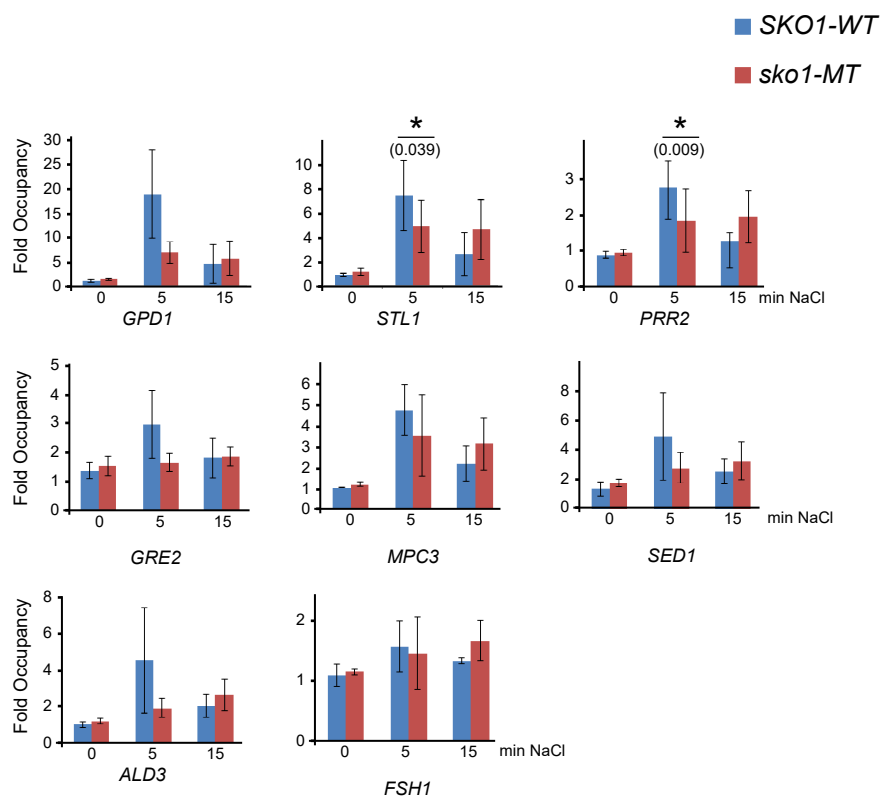

Supplement: S6 Fig — ChIP-qPCR analysis of Hog1.Myc occupancy at indicated genes in SKO1-WT and sko1-MT strains at 0, 5, or 15 min after treatment with NaCl. Data are represented as fold occupancy (relative to occupancy at the PMA1 locus which is not targeted by Hog1 or Sko1). Error bars represent standard deviations of three independent replicates. Asterisks (*) indicate that the compared data pairs are statistically different (P < 0.05; see Materials and Methods). Statistical comparison of Hog1.Myc recruitment is shown in Fig 6E. (PDF) [file pgen.1007991.s006.pdf]
