# Supplementary material for: Sumoylation of DNA-bound transcription factor Sko1 prevents its association with nontarget promoters
Source: PLoS Genet. 2019 Feb 14;15(2):e1007991. doi: 10.1371/journal.pgen.1007991 (PMC6392331; doi:10.1371/journal.pgen.1007991)

Supplementary Figure S4

A

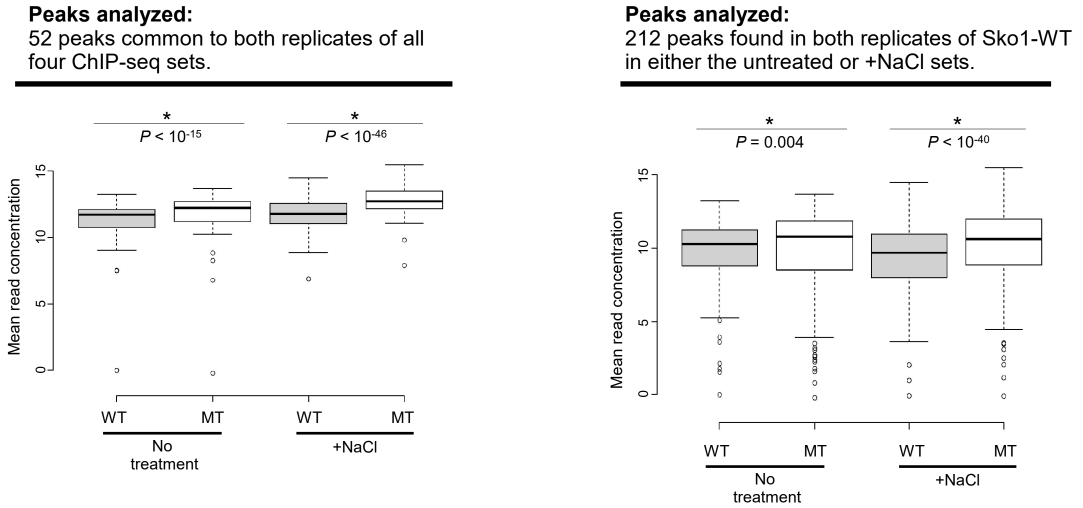

B

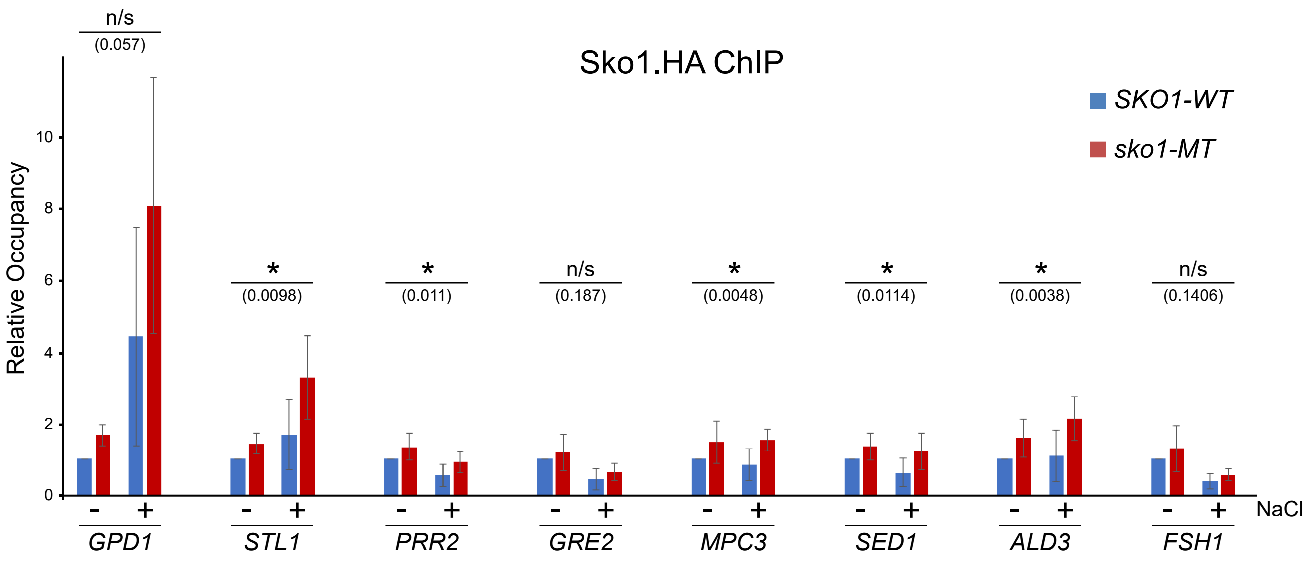

Supplement: S4 Fig — (A) Differential binding analysis of different groups of peaks. Boxplots comparing mean read concentrations (log2 normalized ChIP read counts) in each of the four indicated ChIP-seq analyses for the 52 peaks that are found in both replicates of all four ChIP-seq sets (left) or the 212 peaks that are found in both replicates of the Sko1-WT sets in both untreated and +NaCl conditions (right). (B) Validation of ChIP-seq analysis. Five independent standard ChIP experiments were performed with SKO1-WT and sko1-MT strains. Sko1.HA occupancy levels at promoter regions of eight representative genes were determined by qPCR, at 0 or 5 min after the addition of 0.4 M NaCl. For each gene, occupancy is shown relative to Sko1-WT in untreated samples. Error bars represent standard deviations. P-values from two-factor ANOVA analysis of WT vs MT sets for each gene are shown. Asterisks (*) indicate that the two data sets (WT and MT) are statistically different (P < 0.05; see Materials and Methods). (PDF) [file pgen.1007991.s004.pdf]
