# Supplementary material for: Sumoylation of DNA-bound transcription factor Sko1 prevents its association with nontarget promoters
Source: PLoS Genet. 2019 Feb 14;15(2):e1007991. doi: 10.1371/journal.pgen.1007991 (PMC6392331; doi:10.1371/journal.pgen.1007991)
Supplement: S1 Table — (DOCX) [file pgen.1007991.s007.docx]

**Supplementary Table S1. Yeast strains used in this study**

| **Strain** | **Parental** | **Genotype** |
| --- | --- | --- |
| ***Background Strain*** | | |
| W303a |  | *MAT a ura3-52 trp1Δ2 leu2-3_112 his3-11 ade2-1 can1-100* |
| HF7c |  | *MAT a ura3-52, his3-200, lys2-801, ade2-101, trp1-901, leu2-3, 112,*  *gal4-542, LYS2::GAL1UAS-GAL1TATA-HIS3,*  *URA3::(GAL 17mers) 3-Cyc1TATA-lacZ* |
| ***Derived Strains*** | | |
| YVS003C | W303a | *SKO1-6HA::kl TRP1* |
| YVS007K | W303a | *sko1-K567R-6HA::KlTRP1* |
| YVS008A | W303a | *sko1Δ::kanMX* |
| YVS035B | W303a | *sko1-K450E-6HA:: KlTRP* |
| YVS036B | W303a | *sko1-Δ(445-456)-6HA:: KlTRP* |
| YVS052A | HF7c | *[pGAL4DBD-Sko1-K450E-6HA]* |
| YVS047B | W303a | *sko1-S108,126A,T113A-6HA:: KlTRP* |
| YVS051E | W303a | *sko1-S380,393,399A-6HA:: KlTRP* |
| YVS041A | YVS003C | *SKO1-6HA::KlTRP hog1 Δ:: KanMX* |
| YVS011A | YVS003C | *SKO1-6HA:KlTRP1 HOG1-3MYC::KanMX6* |
| YVS021A | YVS007K | *sko1-K567R-6HA::KlTRP1 TUP1-3MYC::KanMX6* |
