# Supplementary material for: Sumoylation of DNA-bound transcription factor Sko1 prevents its association with nontarget promoters
Source: PLoS Genet. 2019 Feb 14;15(2):e1007991. doi: 10.1371/journal.pgen.1007991 (PMC6392331; doi:10.1371/journal.pgen.1007991)
Supplement: S2 Table — (DOCX) [file pgen.1007991.s008.docx]

**Supplementary Table S2. Oligonucleotide primers used in this study.**

| **Gene** | **Oligonucleotide sequence(s)** |
| --- | --- |
| ***Primers for quantitative PCR analysis of ChIP samples*** | |
| *RTC3* promoter | Forward: 5′- AAGATTTCCCGTTGCGCTAT -3′ |
|  | Reverse: 5′- GGAGAAGAGACACGGAGTAGGA -3′ |
| *ENA1* promoter | Forward: 5′- GGTCTTAAACATCGCCGTGC -3′ |
|  | Reverse: 5′- CCCTGCCCTAACAAACGTCA-3′ |
| *PMA1* promoter | Forward: 5′- CAATTATGACCGGTGACGAAAC -3′ |
|  | Reverse: 5′- AATCGAAACTAATGGAGGGGAG -3′ |
| *GPD1* promoter | Forward: 5′-CCCACCCACACCACCAATAC -3′ |
|  | Reverse: 5′-CCCATTCTGATACTTGTTGTGC -3′ |
| *STL1* promoter | Forward: 5′-TTGTCCCACTATTCCACCGC -3′ |
|  | Reverse: 5′-GGACAAAGTCGGACCCTTCA -3′ |
| *PRR2* promoter | Forward: 5′-ATCGGAGCTACTTTTCCGCA -3′ |
|  | Reverse: 5′-CCCCAATATGCTAACAGCCG -3′ |
| *GRE2* promoter | Forward: 5′-AACAATTGGCCCTCACCTCT -3′ |
|  | Reverse: 5′-ACTTCCGCGAGAAAATTCCGTA -3′ |
| *MPC3* promoter | Forward: 5′-CCCGCTTTTATTTCTCCCGC -3′ |
|  | Reverse: 5′-GAGCCTTTCGGTTTTGCGTG -3′ |
| *SED1* promoter | Forward: 5′-ACCACTGATTGCTCCACGTC -3′ |
|  | Reverse: 5′-AATGACCGTGTGTGCTCTGG -3′ |
| *ALD3* promoter | Forward: 5′-CTGCATATGACGTCTGTTCTTC -3′ |
|  | Reverse: 5′-AAATGCACTAAAGGGCGTGG -3′ |
| *FSH1* promoter | Forward: 5′-CGCCGTATGCATGGGATGAT -3′ |
|  | Reverse: 5′-TCTTTGGCCTATGCGTGTTG -3′ |
| ***Primers for quantitative RT-PCR analysis*** | |
| *STL1* | Forward: 5′-TGAAACCGCCGGAAGAAGTT -3′ |
|  | Reverse: 5′-CCATGGTTGAGTGCCATCCT -3′ |
| *PRR2* | Forward: 5′-CACCAGGAAAGCACAGTTGC -3′ |
|  | Reverse: 5′-TGATCCAGTTGAGACTGGCG -3′ |
| *MPC3* | Forward: 5′-CCAACTTTGAAGTGGGGGCT -3′ |
|  | Reverse: 5′-TGATGACAAACGACCAACGC -3′ |
| *FSH1* | Forward: 5′-GGTGCCGCATTGTCCTCTAT -3′ |
|  | Reverse: 5′-CCAGGATGTTCTGGGTCTGG -3′ |
| *ACO1* | Forward: 5′-TGGTGTTGACACCTTCTCCG -3′ |
|  | Reverse: 5′-TACCACGACCAGTTGCTTCC -3′ |
| *WTM1* | Forward: 5′-TGAAGCTGCCACCACTGATT -3′ |
|  | Reverse: 5′-GTCGACAACAGAATCACCGC -3′ |
| *SAH1* | Forward: 5′-CGGTGATGTCGGTAAGGGTT -3′ |
|  | Reverse: 5′-TCGGTAACCAAGACACGAGC -3′ |
| *PFK2* | Forward: 5′-CCGCTTCTTCAACCAGAGGT -3′ |
|  | Reverse: 5′-TGAGCACCAACAGCCAAAGA -3′ |
| *TDA6* | Forward: 5′-CGGAAACGCATATCCCCAGT -3′ |
|  | Reverse: 5′-GCGCCTTAGAGTTGTAGCCT -3′ |
| *GIT1* | Forward: 5′-AGCAACCGCTGTTAGAGGTG -3′ |
|  | Reverse: 5′-GGGTTGGAAACATTCGACGC -3′ |
| *BSC1* | Forward: 5′-CTGACGGTTGCACAGTTTGG -3′ |
|  | Reverse: 5′-AATCGAAGGTGGTTGTCCCC -3′ |
| *CDC6* | Forward: 5′-ATGGCCATCAAATTCGCAGC -3′ |
|  | Reverse: 5′-AGGTCACCCGTATTTCCAGC -3′ |
| *25S* | Forward: 5′- TCTAGCATTCAAGGTCCCATTC -3′ |
|  | Reverse: 5′- CCCTTAGGACATCTGCGTTATC -3′ |
